# Synthesis of Novel Plant-Derived Encapsulated Radiolabeled Compounds for the Diagnosis of Parkinson’s Disease and the Evaluation of Biological Effects with In Vitro/In Vivo Methods

**DOI:** 10.1007/s12035-024-04103-w

**Published:** 2024-04-03

**Authors:** Emre Uygur, Kadriye Büşra Karatay, Emine Derviş, Vedat Evren, Ayfer Yurt Kılçar, Özge Kozguş Güldü, Ceren Sezgin, Burcu Acar Çinleti, Volkan Tekin, Fazilet Zumrut Biber Muftuler

**Affiliations:** 1 Soma Vocational School, Department of Biomedical Device Technologies, Manisa Celal Bayar University, Nihat Danışman, Değirmen Cd. No. 2, Soma, 45500 Manisa, Turkey; 2 Institute of Nuclear Sciences, Ege University, Erzene, Ege Üniversitesi, Ege Ünv., 35100 Bornova, İzmir Turkey; 3 Faculty of Medicine, Department of Physiology, Ege University, Bornova, 35100 İzmir Turkey; 4 Department of Nuclear Medicine, Manisa City Hospital, Adnan Menderes Neighborhood, 132Nd Street Number 15 Şehzadeler, 45100 Manisa, Turkey; 5 Faculty of Medicine, Buca Seyfi Demirsoy Training and Research Hospital, Department of Neurology, Izmir Democracy University, Kozağaç Mah. Özmen Cad. No. 147, Buca, 35040 Izmir Turkey

**Keywords:** [^99m^Tc]Tc, Madecassoside (MA), L-DOPA, Parkinson’s disease, PLGA, Encapsulation, In vitro, In vivo

## Abstract

Parkinson’s disease (PD) is a neurodegenerative disorder that affects millions of individuals globally. It is characterized by the loss of dopaminergic neurons in Substantia Nigra pars compacta (SNc) and striatum. Neuroimaging techniques such as single-photon emission computed tomography (SPECT), positron emission tomography (PET), and magnetic resonance imaging (MRI) help diagnosing PD. In this study, the focus was on developing technetium-99 m ([^99m^Tc]Tc) radiolabeled drug delivery systems using plant-derived compounds for the diagnosis of PD. Madecassoside (MA), a plant-derived compound, was conjugated with Levodopa (L-DOPA) to form MA-L-DOPA, which was then encapsulated using Poly Lactic-co-Glycolic Acid (PLGA) to create MA-PLGA and MA-L-DOPA-PLGA nanocapsules. Extensive structural analysis was performed using various methods such as Fourier-transform infrared spectroscopy (FTIR), nuclear magnetic resonance spectroscopy (NMR), liquid chromatography–mass spectrometry (LC–MS), thin layer chromatography (TLC), high performance liquid chromatography (HPLC), dynamic light scattering (DLS), and scanning electron microscopy (SEM) to characterize the synthesized products. Radiochemical yields of radiolabeled compounds were determined using thin layer radio chromatography (TLRC) and high performance liquid radio chromatography (HPLRC) methods. In vitro cell culture studies were conducted on human neuroblastoma (SH-SY5Y) and rat pheochromocytoma (PC-12) cell lines to assess the incorporation of [^99m^Tc]Tc radiolabeled compounds ([^99m^Tc]Tc-MA, [^99m^Tc]Tc-MA-L-DOPA, [^99m^Tc]Tc-MA-PLGA and [^99m^Tc]Tc-MA-L-DOPA-PLGA) and the cytotoxicity of inactive compounds (MA and MA-L-DOPA compounds and encapsulated compounds (MA-PLGA and MA-L-DOPA-PLGA). Additionally, the biodistribution studies were carried out on healthy male Sprague–Dawley rats and a Parkinson’s disease experimental model to evaluate the compounds’ bioactivity using the radiolabeled compounds. The radiochemical yields of all radiolabeled compounds except [^99m^Tc]Tc-L-DOPA-PLGA were above 95% and had stability over 6 h. The cytotoxic effects of all substances on SH-SY5Y and PC-12 cells increase with increasing concentration values. The uptake values of PLGA-encapsulated compounds are statistically significant in SH-SY5Y and PC-12 cells. The biodistribution studies showed that [99mTc]Tc-MA is predominantly retained in specific organs and brain regions, with notable uptake in the prostate, muscle, and midbrain. PLGA-encapsulation led to higher uptake in certain organs, suggesting its biodegradable nature may enhance tissue retention, and surface modifications might further optimize brain penetration. Overall, the results indicate that radiolabeled plant-derived encapsulated drug delivery systems with [^99m^Tc]Tc hold potential as diagnostic agents for PD symptoms. This study contributes to the advancement of drug delivery agents in the field of brain research.

## Introduction

Parkinson’s disease (PD) is the second most common neurodegenerative disease after Alzheimer’s disease, affecting about six million people worldwide [1–3]. The risk of developing PD increases with age, particularly between the ages of 65 and 90. PD is more common in male patients [4]. According to a research, the total number of patients with PD are estimated to increase from 8.7 to 9.3 million by 2030 [5]. Currently no cure or preventive treatment exists for PD [4, 6, 7]. The cardinal symptoms of PD are tremor, rijidity, bradykinesia, and postural instability [5]. In clinical practice, the diagnosis of PD is based on clinical features. Three levels of diagnostic confidence are differentiated: Definite, Probable, and Possible. The diagnoses of Possible and Probable PD are solely based on clinical criteria. Neuropathologic confirmation is required for the diagnosis of Definite PD [1–7]. PD is pathologically characterized by the loss of dopaminergic neurons in the Substantia Nigra pars compacta (SNc) and striatum regions of the brain. Another key pathological hallmark of PD is the presence of Lewy bodies, which are protein aggregates that accumulate in the cytoplasms of cells [8]. Symptoms occur when at least 50% of dopamine-nigral cells are lost [3–5]. As the loss of dopaminergic neurons continues, the disease progresses [9, 10]. Therefore, early diagnosis of the disease is of great importance. Molecular neuroimaging studies (SPECT or PET) have previously demonstrated that radioligands with high affinity to the dopaminergic system (dopamine transporter-DAT, DOPA decarboxylase activity, and vesicular monoamine transporters) estimate the dopamine cell loss and may be used as a surrogate marker of PD, in the prodromal, pre-motor, preclinical phase [6]. In recent years, there has been an increase in research on the potential useof herbal compounds in the diagnosis and treatment of Parkinson's disease [1, 4, 11–16]. In these studies, the antioxidant properties of plants [17], MAO-B enzyme inhibitory properties [12], anti-inflammatory effects, and neuroprotective properties [13, 18, 19] come to the fore. Particularly, considering the destruction of dopaminergic neurons as the cause of PD, compounds that exert antioxidant properties and neuroprotective effects on these neurons have become more important [20].

One of these medicinal plants is Centella Asiatica (CA), which contains compounds that exhibit neuroprotective effects in PD. In addition to the neuroprotective effect of CA, it has been reported by many studies in the literature that it has a wide range of biological activities such as wound healing, anti-inflammatory, antiulcer, hepatoprotective, anticonvulsant sedative, immunostimulant, cardioprotective, antidiabetic, cytotoxic, antitumor, antiviral, antibacterial, and insecticidal [15, 16, 21, 22]. CA contains asiaticoside, madecassoside, madecassic acid, and asiatic acid [23, 24]. Madecassoside (MA) is a triterpenoid saponin derived from the CA plant and is reported to be the highest component of the major bioactive triterpene components in CA [22]. MA, which has a pharmacological effect on the central nervous system, has neuron-protective properties, especially on dopaminergic cells in the SNc and striatum regions of the brain [13, 25].

Levodopa (L-DOPA, L-3,4-dihydroxyphenylalanine) is the precursor to dopamine. Currently, Exogenous Levodopa is used as a dopamine replacement agent to reduce the symptoms of PD. The efficacy of exogenous levodopa (L-DOPA) is attributed to its conversion to dopamine by the enzyme aromatic L-amino-acid decarboxylase in striatal dopaminergic terminals [26]. It is well established that L-DOPA has high specificity for dopaminergic neurons in the SNc [27, 28]. The advances in drug delivery systems with nanoparticles, which may be used in various conditions such as vaccination, cancer, inflammation, and other disease, have made it more valuable as a research and therapeutic subject in the past decade [29]. Particularly in drug delivery mechanisms using NPs, polymeric particulate structures protect the active substance from degradation, to get integrated into a solid matrix, increasing the possibility of the substance reaching the brain [29–31]. Biodegradable polyester NPs such as poly-lactic-co-glycolic acid (PLGA) have been widely used in a variety of drugs due to their controlled biodegradability, excellent biocompatibility, and high safety in biomedical applications. PLGA is one of the few biodegradable synthetic polymers because of its biodegradation kinetics, non-toxic effects, and mechanical properties [29, 32]. The mechanism of cellular uptake of nanoparticles with PLGA isendocytosis and the drug is released at the targeted site afterwards. Radiolabeled polymeric nanoparticles (NPs) can be produced through methods such as surface coupling, encapsulation, isotope exchange, non-isotope exchange, and PEGylation (polyethylene glycol attachment) [33, 34].

Technetium-99 m ([^99m^Tc]Tc) is an easy, convenient, low-cost radionuclide used in the development of diagnostic kits in nuclear medicine, with an ideal imaging energy (140 keV) and and short half-life (t_1/2_ = 6 h) [35]. [^99m^Tc]Tc can bind to many different compounds; therefore, approximately 85% of imaging procedures in nuclear medicine are performed with compounds radiolabeled with [^99m^Tc]Tc [36]. In nuclear medicine, radionuclides can be directed to targeted tissues with molecules that are selective for those tissues and organs. There are many scientific articles in the literature that examine the bioactivities of plant-derived radiolabeled compounds on specific organs and tissues [34, 37].

The current study aimed to develop radiolabeled plant-origin-encapsulated drug delivery systems with [^99m^Tc]Tc for the diagnosis of PD. For this purpose, L-DOPA conjugation (MA-L-DOPA) of MA, a plant-based compound, was made and the conjugate was encapsulated with PLGA (MA-PLGA and MA-L -DOPA-PLGA). All compounds were radiolabeled with [^99m^Tc]Tc. The cytotoxicity studies with inactive (MA, MA-L-DOPA, MA-PLGA, and MA-L-DOPA-PLGA) compounds and incorporation studies with radiolabeled compounds were performed on SH-SY5Y and PC-12 cell lines. In order to investigate the bioactivity of [^99m^Tc]Tc radiolabelled compounds, biodistribution studies were carried out on healthy male Sprague–Dawley rats and experimental model of PD created by stereotaxic intervention in male Spraque-Dawley rats.

## Experimental

### Chemicals and Reagents

Madecassoside, L-3,4-Dihydroxyphenylalanine (Levodopa, L-DOPA), Rotenone, and Dimethyl Sulfoxide (DMSO) were purchased from Sigma Aldrich co. Thin-layer chromatography paper (ITLC-cellulose/ silica), ethanol, methanol, n-octanol, n-butanol, isopropyl alcohol, and acetonitrile were purchased from Merck Chemical Co., min. the essential amino acid, Dulbecco’s MEM, Min. Essential Medium (Mem Eagle), RPMI 1640 Medium, L-glutamine, sodium bicarbonate, sodium pyruvate, fetal bovine serum, penicillin/streptomycin, trypan blue, phosphate buffer solution, trypsin ethylenediaminetetraacetic acid (EDTA) were purchased from Biological Industries. Technetium-99 m (Na[^99m^Tc]TcO4) was supplied from Monrol co. (Eczacibasi, Monrol, Istanbul, Turkey).

### Synthesis of MA-L-DOPA

**Fig. 1:**
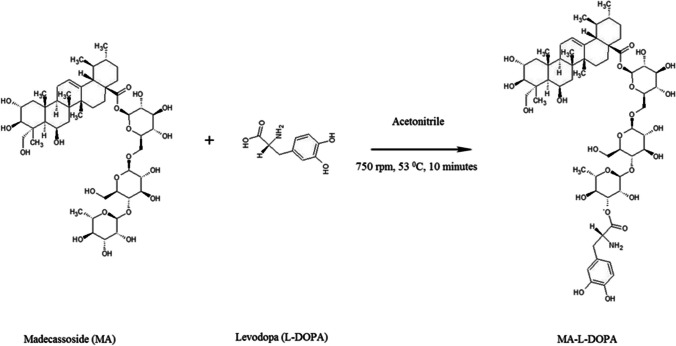
The scheme of the synthesis of MA-L-DOPA

The esterification reaction was carried out by an optimized method according to Şenocak's (2010) study [38]. Accordingly, 0.025 mmol (2.5 mg) MA and 0.025 mmol (0.5 mg) L-DOPA were taken into a tube and 5 mL acetonitrile was added as a solvent. The mixture was vortexed. The conjugate obtained (MA-L-DOPA, Fig. 1) was mixed in a magnetic stirrer at 750 rpm and 53 ^0^C for 10 min. After 10 min, white-colored precipitates were observed in the solution. The centrifugation was performed at 4000 rpm for 15 min to separate the precipitate and the upper phase was separated with a pipette. Acetonitrile was evaporated by placing both tubes containing the upper and lower phase in the oven.

### Structural Analysis of MA-L-DOPA

The following analyses were performed to verify the structural analysis of the MA-L-DOPA synthesis, respectively. Melting Point Determination (Electrothermal 9200), Fourier Transform Infrared Spectroscopy (FTIR, Perkin Elmer Spectrum BX II) spectra were obtained at Afyon Kocatepe University Technology Faculty Laboratories. Nuclear Magnetic Resonance (^1^H-NMR, Bruker Ultrashield 300 MHz), Liquid Chromatography / Mass Spectrometry (LC / MS, Waters 2695 Alliance Micromass ZQ) analyzes were performed at Ankara University, Faculty of Pharmacy Central Laboratory. In addition, the expected theoretical NMR spectrum for MA-L-DOPA was created using the Advanced Chemistry Development (ACD) computer program to compare with the experimental NMR spectrum.

### Thin Layer Chromatography (TLC)

MA and L-DOPA were dissolved in methanol (1 mg / mL). On the other hand, the MA-L-DOPA was dissolved in 100 µL of methanol since its synthesis product is less. Silica gel TLC papers (1.2–10 cm sizes) and the methanol–water (9: 1) solvent system was prepared. The samples (six samples, two from each sample) were dropped 0.5 cm away from the paper ends, and the papers were placed in the TLC tank. The papers removed from the TLC tank were allowed to dry and the dried papers were observed under the UV lamp. Relative Front (R_f_) values of each sample were calculated.

### High-Performance Liquid Chromatography (HPLC)

HPLC analysis was performed with Shimadzu LC-10Atvp (SPD-10AV) HPLC system with ODS 5- µm C18 RP-C18 (250 × 4.6 mm ID) (GL Sciences Inc.) column. HPLC system was conditioned as 20:80 (v / v) methanol–water mobile phase, flow rate 1 mL/ min, wavelengths (205–280 nm). The MA-L-DOPA, MA and L-DOPA molecules were analyzed under the same conditions. The retention times (R_t_) values of the samples were determined in the chromatograms obtained.

### Encapsulation of MA and MA-L-DOPA

The conditions were optimized according to Peltonen et al. [39]. Briefly, 500 µL DMSO was added to 10 mg PLGA and 500 µL DMSO was added to 2 mg MA. The obtained MA solution was added to the PLGA solution and the mixture was taken into a syringe. Ten milliliters of distilled water was placed in a beaker and mixed at room temperature in such a way that a vortex was formed in the magnetic stirrer. The mixture in the syringe was added dropwise to the pure water in the mixer. The final solution was stirred on a magnetic stirrer for 30 min. The solution was centrifuged at 10,000 rpm for 10 min and the upper and lower phases were separated. The same procedure was done for MA-L-DOPA**.**

### Characterization Studies of MA-L-DOPA-PLGA

DLS (Malzem Zeta Sizer) analysis of the samples (30 µg/mL in methanol, dispersant refractive index: 1.330, viscosity (cP):0.8872) were taken from the upper and lower phase was performed. The same procedure was performed for MA-L-DOPA-PLGA. The SEM (Thermo Scientific Apreo S) images of the samples (MA-PLGA and MA-L-DOPA-PLGA) were taken at Ege University Center Research Test and Analysis Laboratory Application and Research Center.

### Radiolabeling

Radiolabeling conditions with [^99m^Tc]Tc radioisotope have been optimized by researchers. Different pH (pH 3, 5, 7, 9, and 12) values have been tested within the scope of optimization studies. The best result of pH is 12. In addition, different solvent systems (saline solution, acetonitrile, 9:1 (v/v) methanol–water, 2:8 (v/v) methanol–water) were also investigated. The best solvent system was 2:8 (v/v) methanol–water. Accordingly, MA, L-DOPA, and MA-L-DOPA (1 mg / mL) were dissolved in distilled water. Respectively, 25 µg MA, 25 µg SnCl_2_.2H_2_O (Tube-1), 25 µg L-DOPA, 25 µg SnCl_2_.2H_2_O (Tube-2), and 15 µg of MA-L-DOPA, 35 µL of distilled H_2_O, 25 µg of SnCl_2_.2H_2_O (Tube-3) solutions were placed in different tubes. For encapsulated compounds, MA-PLGA and MA-L-DOPA-PLGA (1 mg / mL) were dissolved in DMSO. 30 µg MA-PLGA, 70 µL DMSO, 25 µg SnCl_2_.2H_2_O (Tube 4) and 30 µg MA-L-DOPA-PLGA, 70 µL DMSO, 25 µg SnCl_2_.2H_2_O (Tube 5) solutions were placed in different tubes. The pH value was brought to 12 with 0.1 M NaOH for each tube. 22.2 MBq (600 µCi) (Na[^99m^Tc]TcO4) was added to each tube. Thirty minutes of incubation was performed in room conditions (25 ^0^C). Quality control studies of radiolabeled compounds were carried out by using TLRC and HPLRC methods.

### Thin Layer Radio Chromatography (TLRC) Procedure

The aluminum (ITLC-SG) sheets covered with silica gel (size, 1.50 × 10 cm- thick, 0.1 mm), and methanol–water (2:8, v/v) solvent system. All radiolabeled compounds ([^99m^Tc]Tc-MA, [^99m^Tc]Tc-L-DOPA, [^99m^Tc]Tc-MA-L-DOPA, [^99m^Tc]Tc-MA-PLGA and [^99m^Tc]Tc-MA-L-DOPA-PLGA) were dropped on the prepared strips 0.50 cm above the base counted on the TLRC Scanner (Bioscan AR2000).

### High-Performance Liquid Radio Chromatography (HPLRC) Procedure

HPLRC analyses were performed under the same conditions as the method applied for inactive ingredients in the HPLC procedure. Differently, the radioactivity of the components ([^99m^Tc]Tc-MA, [^99m^Tc]Tc-L-DOPA, [^99m^Tc]Tc-MA-L-DOPA) was detected using the NaI (Tl) detector (Gabi Star, Raytest) in the HPLC system.

### Stability Studies

To determine the stability, all radiolabeled compounds were applied to silica strips as in the TLRC procedure (Sect. 2.8) at different times (0, 30, 60, 90, 120, and 240 min). Radiochemical yields at these times were examined by the TLRC method and the change over time was examined.

### Lipophilicity Studies

0.3 mL of n-octanol and 0.3 mL of pH = 7 buffer were placed in a centrifuge tube, then 0.1 mL of the radiolabeled compound was added and the whole mixture was vortexed for 1 min. It was centrifuged at 2500 rpm for 30 min to separate the upper and lower phases. One hundred microliter samples were taken from each of these phases and counts were taken in the Cd (Te) detector. At the same time, the experimental lipophilicity values obtained were compared with the theoretical lipophilicity values obtained from the ACD / Labs logP Algorithm program (Version 6.0).

### In Vitro Cell Culture Studies

 SY-SH5Y and PC-12 cell lines were used in in vitro cell culture studies. SH-SY5Y cells in a medium consisting of minimum Essential Medium (Eagle) and 10% fetal bovine serum (FBS); PC-12 cells were produced in a medium consisting of RPMI 1640 medium, 10% horse serum, and 5% fetal bovine serum (FBS). All cells were incubated in 5% CO_2_ and 37 °C. Fresh medium was added by changing the medium every 2 days. After the cells were produced enough to cover 80% of the flasks, they were separated from the flask by means of a 0.25% (W / V) trypsin–EDTA solution and plated in 24-well plates for incorporation studies and 96-well plates for cytotoxicity studies and study groups were formed. Cytotoxicity studies of inactive compounds and incorporation studies of all radiolabeled compounds were carried out on both cell lines.

### Cytotoxicity Studies

Cells were prepared from SY-SH5Y and PC-12 cell suspensions at 5 × 10^4^ cells / mL in each well of 96-well plates. One hundred microliters of cell suspension was added to each well-formed and the solution containing MA prepared with sterile SF at five different (10^0^, 10^–1^, 10^–2^, 10^–3^, 10^–4^ nM) concentrations was added to the wells outside the control. As a negative control, media without cells and reagents were used. In the study, each parameter was studied in triplicate. The plate containing the cells was incubated at 37 °C in 5% CO_2_. SH-SY5Y cells were incubated at 24, 48, and 72 h of incubation by adding 10 μl of WST solution to each well and incubating for another 4 h. On other hand, MTT solution was added to PC-12 cells and incubated for 4 more hours. At the end of the incubation, the MTT solution on the cells was removed and 200 µL DMSO was added to the PC-12 cells. All cells were read using a spectrophotometer, at 450 nm wavelength and 690 nm reference range, for the absorbance value of each well. Negative control was accepted as zero absorbance and % viability values were calculated with the formula [(measured absorbance value/control value) × 100] and the same procedures were repeated for the MA-L-DOPA compound.

### Incorporation Studies

SH-SY5Y cells were taken into 24-well plates and PC-12 cells were taken into eppendorf with 0.5 mL of medium. The time parameters were determined as 30, 60, 120, and 240 min. For SY-SH5Y cells, the existing medium was removed, and a ^99m^Tc-free medium was placed on the cells in the 24-well plate as the control group. Each well was washed with saline (SF). Medium containing 0.5 mL of radiolabelled 1 mCi (37 MBq) compounds ([^99m^Tc]Tc-MA, [^99m^Tc]Tc-MA-L-DOPA, [^99m^Tc]Tc-MA-PLGA and [^99m^Tc]Tc-MA-L-DOPA-PLGA), respectively, were added to each well of the plates. Initial radioactivity (A_0_) was determined by counting the radiolabelled medium on the cells in each well at the Cd (Te) detector at 30, 60, 120, and 240 min. In order to examine the effect of the ligands, the same procedure was applied for free [^99m^Tc]Tc. The medium which includes the radiolabelled compound in the well was removed from the cells and the wells were washed with SF. By adding 0.5 mL of SF to each well, the radioactivity count (A_1_) of radiolabeled samples remaining bound to the cells in the wells was repeated.

As the cells were suspended in the PC-12 cell line, cell study groups were formed in Eppendorf instead of 24-well plates. All cells were centrifuged at 2500 rpm for 5 min. After the centrifuge, the existing medium on the cells was removed and 0.5 mL of SF was added to them to remove the dead cells from the environment. They washed again with SF and centrifuge has been done at 2500 rpm for 5 min. After centrifugation, dead cells were removed and the mediums containing 0.5 mL of [^99m^Tc]Tc radiolabelled compounds 1 mCi were added to each eppendorf. The same procedure was applied for free [^99m^Tc]Tc in order to examine the effect of the ligands. Initial radioactivity (A0) was determined by counting the radiolabelled medium on the cells in each Eppendorf at the 30, 60, 120, and 240 min in the CdTe detector. The selected time parameters (30, 60, 120, and 240 min) were kept in an oven at 37 °C. When it is always the turn of the parameter, cells, respectively, 5 min at 2500 rpm. It was centrifuged, after centrifugation, the radiolabelled media on the cells were discarded and the cells removed from the surface were removed by washing them with SF. By adding 0.5 mL of SF to each eppendorf, the radioactivity count (A1) of the radiolabeled samples remaining bound to the cells in the eppendorf was repeated. The % binding values were determined by proportioning the detected A_1_ and A_0_ values and taking the control group counts into consideration. All-time parameters in each cell line were studied in three repeats (*n* = 6) for both cell lines.

### In Vivo Studies

Biodistributions of radiolabelled compounds on Sprague Dawley rats (male, 6–8 weeks, 250–350 g) were investigated within the scope of in vivo studies. Ethics committee permissions required for in vivo studies were obtained with the approval of Ege University Local Animal Experiments Ethics Committee dated 21.12.2016 and numbered 2016–110. According to the literature, the uptake of the MA compound in the brain tissue in rats occurs within 1 h [40]. So, the time parameter set as 60 min. In the study, an experimental Parkinson Model (*n* = 7) was created by using rotenone with a steroid intervention. Healthy Sprague Dawley rats without any intervention were used as a control group (*n* = 12). All experiments were carried out in Ege University, Faculty of Medicine, Department of Physiology.

### Stereotaxic Interference

The conditions were optimized according to Erbas et al. study [41]. The application was made in Ege University Faculty of Medicine, Department of Physiology, Brain Research Laboratory. Briefly, 12 mg / mL rotenone was dissolved in DMSO. The operation was made on seven healthy male Sprague Dawley rats (250–300 g). The anesthetic (2 mL/kg, i.p) agent [2 mL ketamine (80 mg / kg) + 2 mL SF + 1 mL xylazine (4 mg / kg)] was administered to the rats. After anesthesia, the heads of the rats were shaved and placed in a stereotaxic device. The scalp was opened in accordance with the necessary surgical interventions and the bregma was determined. SNc (AP: 5.2 mm, L: 2.4 mm, V: 8 mm) was determined with the help of the rat stereotaxic atlas [42]. Assuming the Bregma origin (0) point 100 µL of rotenone was injected with a 32 Hamilton syringe to the SNc region corresponding to the coordinates. All rats were treated with prophylactic antibiotics to prevent post-surgical infections. After the applications, the health status of the rats was observed for 10 days. During this period, impairment in motor functions and tremor were observed in the rats. End of the 10th day, the rats were injected with apomorphine hydrochloride (2 mg/kg, i.p.) to induce rotational behavior. After administration of apomorphine, the rat’s movements were recorded in a 10-min period. The rats, which turn unilaterally more than 7 cycles/min, were accepted as successful for PD model.

### Biodistribution Studies

The biodistribution study was carried out on Sprague Dawley rats in two stages. In the first stage, determined as the control group (*n* = 12) male Sprague Dawley rats of 4–6 weeks of age and between 250 and 300 g were used. The main applications in the biodistribution study are injection from the tail vein of radiolabeled compounds ([^99m^Tc]Tc-MA, [^99m^Tc]Tc-L-DOPA, [^99m^Tc]Tc-MA-L-DOPA, [^99m^Tc]Tc-MA-PLGA and [^99m^Tc]Tc-MA-L-DOPA-PLGA) extracting tissue samples, weighing, and counting activity in the Cd (Te) detector. The rats were injected to 0.20 mL with an activity value of about 1 mCi per radiolabeled compounds. To determine the net amount of activity injected into the rats, the filled-state activities of the injectors just before the injection and the empty-state activities after the injection were counted in the Cd(Te) detector. After 60 min (the time parameter), the rats were sacrificed under anesthesia (2 mL/kg, i.p, 2 mL ketamine (80 mg / kg) + 2 mL SF + 1 mL xylazine (4 mg / kg). Blood, heart, lung, liver, kidney, small intestine (IB), large intestine (KB), stomach, spleen, pancreas, muscle, testis, prostate, fat, bladder, head, and brain parts of sacrificed rats were removed. The extracted samples were placed in previously tared containers, weighed with sensitive scales, and then activity counts were taken in the Cd(Te) detector. In addition, the cerebellum, hippocampus, medulla pons, striatum, hypothalamus, temporal cortex, midbrain, and frontal cortex parts of the brain were also separated. Brain sections were also placed in previously tared containers, weighed with sensitive scales, and then activity counts were made in the Cd(Te) detector.

In the second stage of the study, Sprague Dawley rats, which underwent stereotaxic surgery to create dopaminergic lesions, were utilized (*n* = 7). In this step, the same procedure in the control group was applied for ([^99m^Tc]Tc-MA, [^99m^Tc]Tc-MA-L-DOPA) compounds based on the data obtained in the first stage. Differently, at this stage, the cerebellum, hippocampus, medulla pons, striatum, hypothalamus, temporal cortex, midbrain, and frontal cortex sections of the brain were divided into right and left (right cerebellum-left cerebellum). Since the right side of the rats was damaged due to stereotactic intervention, both right and left brain regions were examined to determine whether there was a hemispheric difference. The brain sections were placed in containers with tare and weighed with sensitive scales. After weighing the excised brain tissue parts, the radioactivity measurements of these structures were made in the Cd(Te) detector. As a result of these weighing and counting, the activity values per gram (% ID / g) for each structure were calculated in the excel program, considering the time corrections.

### Statistical Analysis

In the analyses, the average binding values and standard deviations were calculated according to a total of six replicates in two separate trials (three replicates for each parameter). While evaluating the statistical analysis results of in vitro and in vivo studies, it was tested whether there was a significant difference at the 95% (*p* < 0.05) confidence level between the intake and uptake values. The *P* values of the statistical results less than 0.05 were accepted as significant difference. One-way analysis of variance (ANOVA) was performed with the Graph Pad program for statistical analysis of the data obtained from in vitro cell culture studies. SPSS 15 statistical program was used for the statistical evaluation of in vivo biodistribution studies. Variance analysis and Pearson correlation statistics were performed for these results.

## Results

### Structural Analysis of MA-L-DOPA

**Fig. 2:**
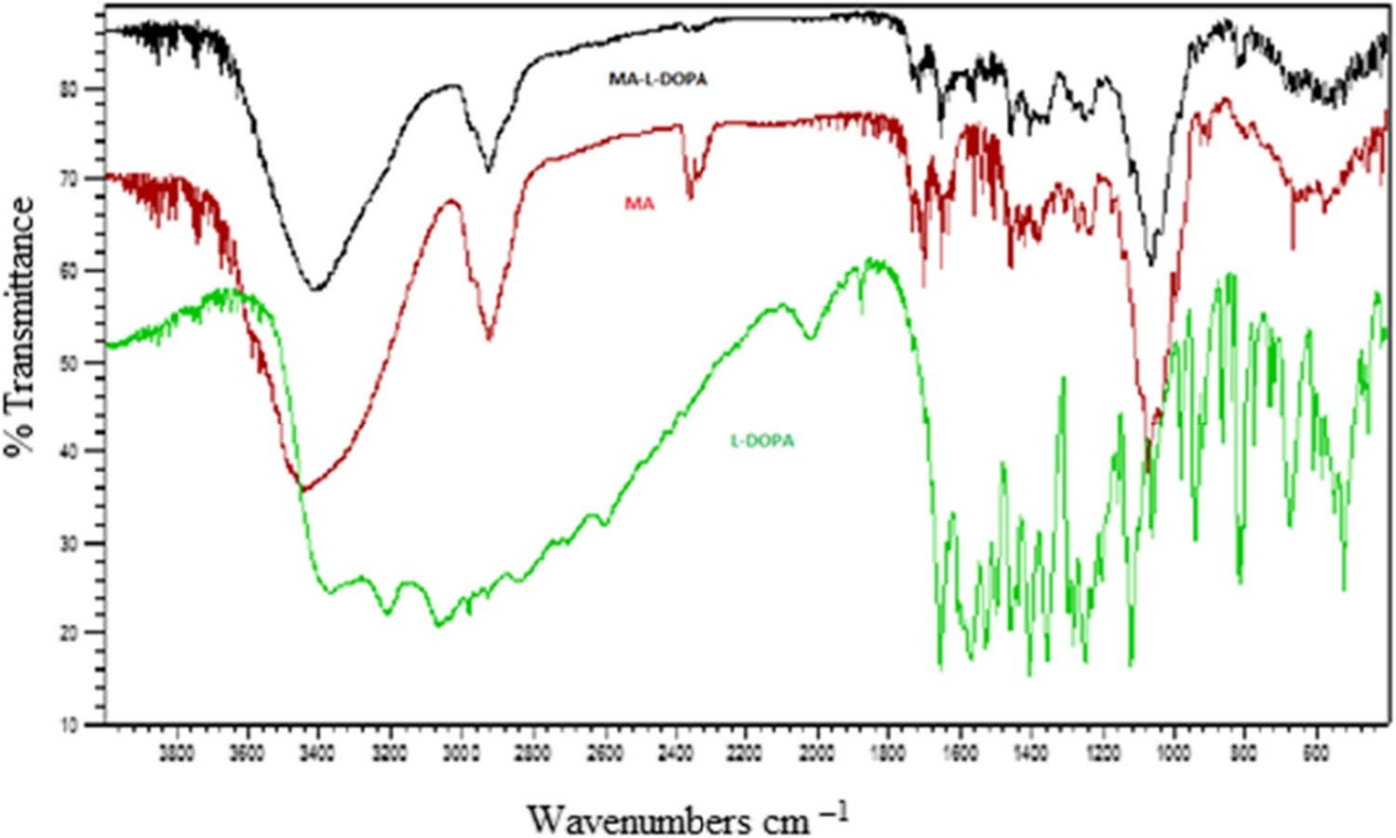
FTIR spectrum of compounds

**Fig. 3:**
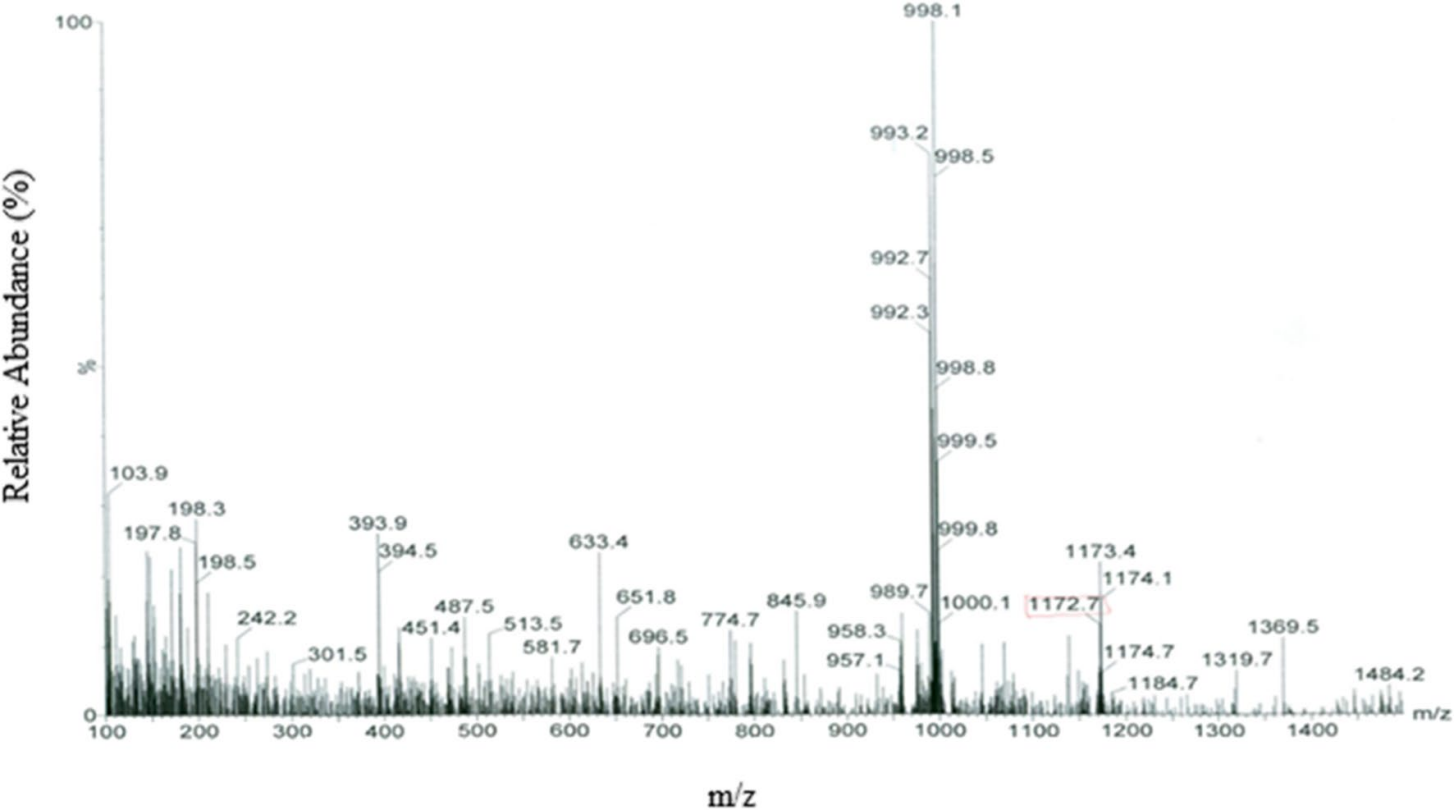
LC/MS spectrum of MA-L-DOPA

**Fig. 4:**
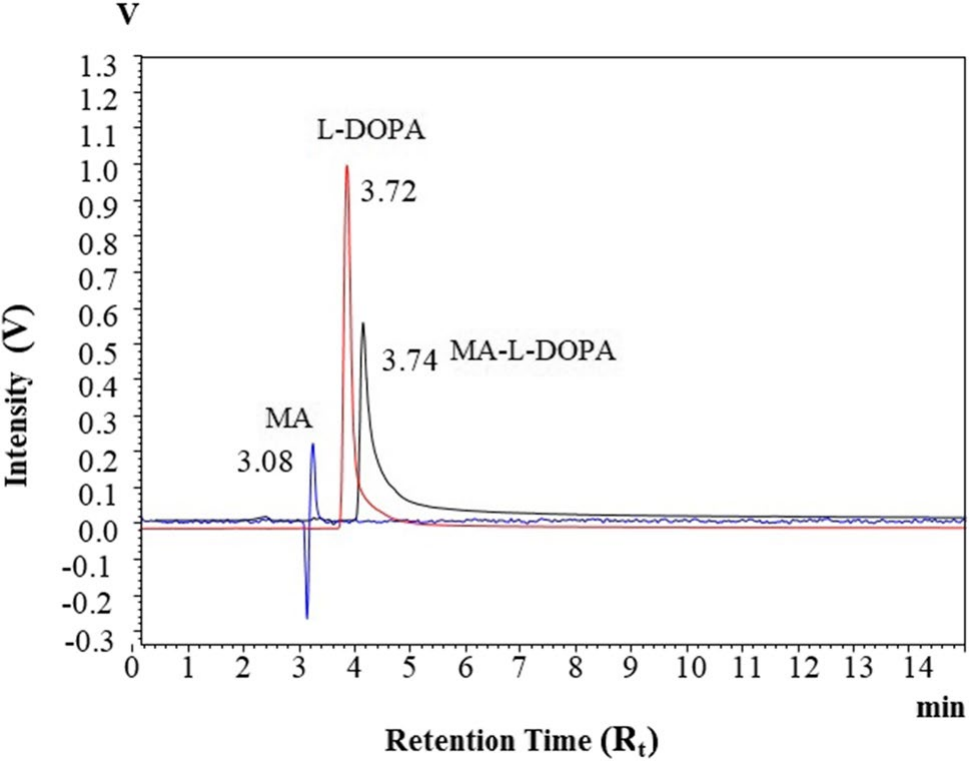
HPLC chromatograms of compounds

The melting point value of the MA-L-DOPA (239–240 ^0^C) compound is different from the melting point of the MA (232–233 ^0^C) and L-DOPA (279–280 ^0^C) compounds. In the FTIR spectrum (Fig. 2), for MA-L-DOPA and MA molecules the –OH bond forms the absorption band at 3000–3600 cm^–1^, but the –OH bond of the L-DOPA molecule has a wider absorption band. The peaks at 1600 cm^–1^ and 1200 cm^–1^ belong to –OH bending and C – O stress bonds, respectively**.** According to the LC/MS analysis **(**Fig. 3), the 998 and 992.3 peaks indicate that sodium and water are attached to the MA molecule [MA + Na]^+^, respectively. MA-L-DOPA molecule peak is the 1172's peak. Theoretical ^1^H-NMR analysis and experimental ^1^H-NMR analysis of MA-L-DOPA compared and the theoretical and experimental ppm results are presented in Table 1. As a result of the TLC analysis, the R_f_ values of MA, L-DOPA, and MA-L-DOPA compounds were 0.97, 0.94, and 0.85, respectively. On the other hand, HPLC chromatograms (Fig. 4) of MA, L-DOPA, and MA-L-DOPA compounds were examined, and retention times (R_t_) of MA, L-DOPA, and MA-L-DOPA were 3.08, 3.72, and 3.74 min, respectively.

**Table 1:** The ppm results in ^1^H-NMR spectra were obtained as a result of theoretical and experimental study of the MA-L-DOPA compound

Carbon number	Theoretical ppm value	Experimental ppm value
31	4.39	4.36
41	6.56	6.61
44	6.76	6.72
45	6.48	6.59
55	2.19	2.24
59	4.23	4.35
64	5.23	5.26
67	4.88	4.89
70	0.95	0.97
72	1.27	1.27
73	1.23	1.25
74	1.78	1.79
77	1.71	1.71

### Characterization of Encapsulated Compounds

**Fig. 5:**
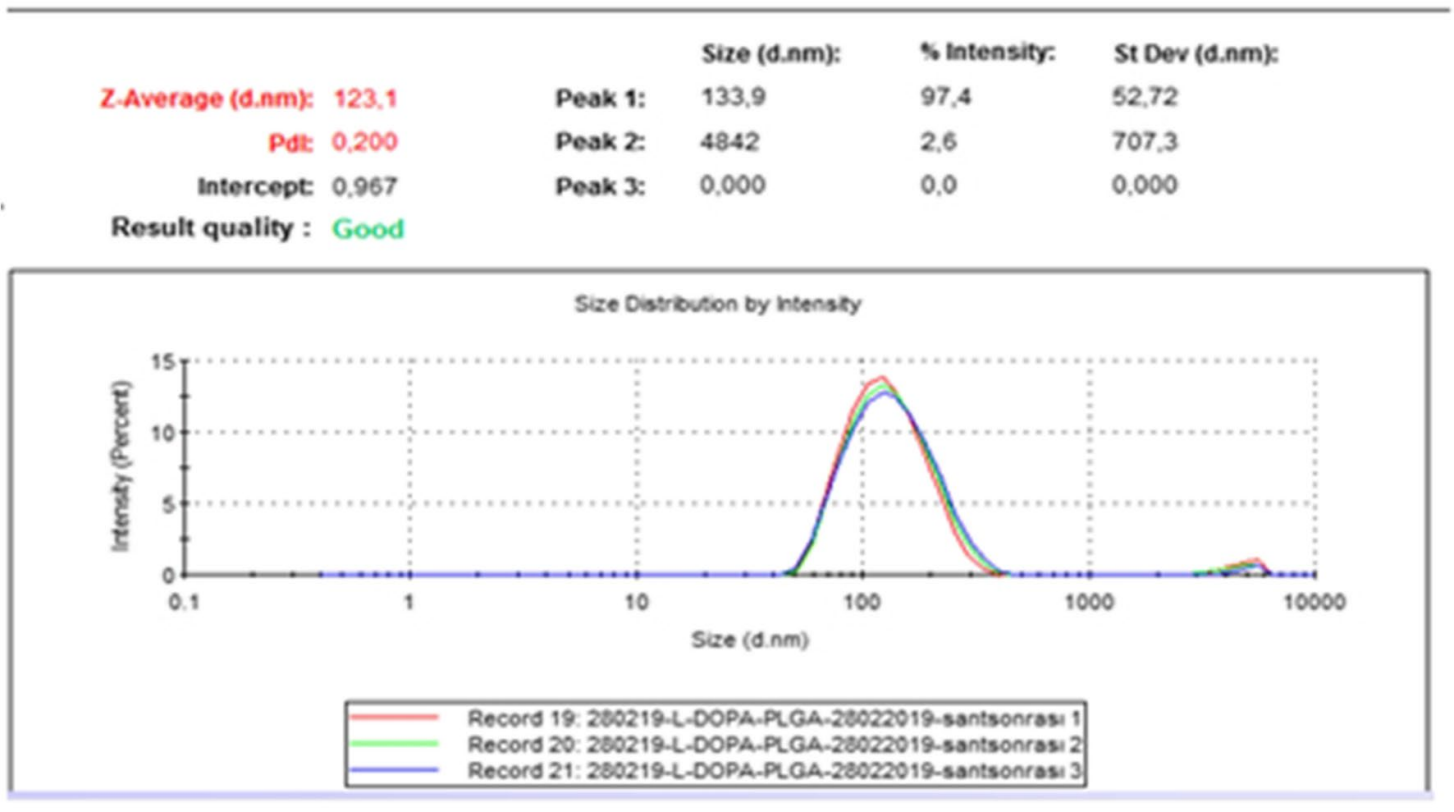
The particle size of L-DOPA-PLGA

**Fig. 6:**
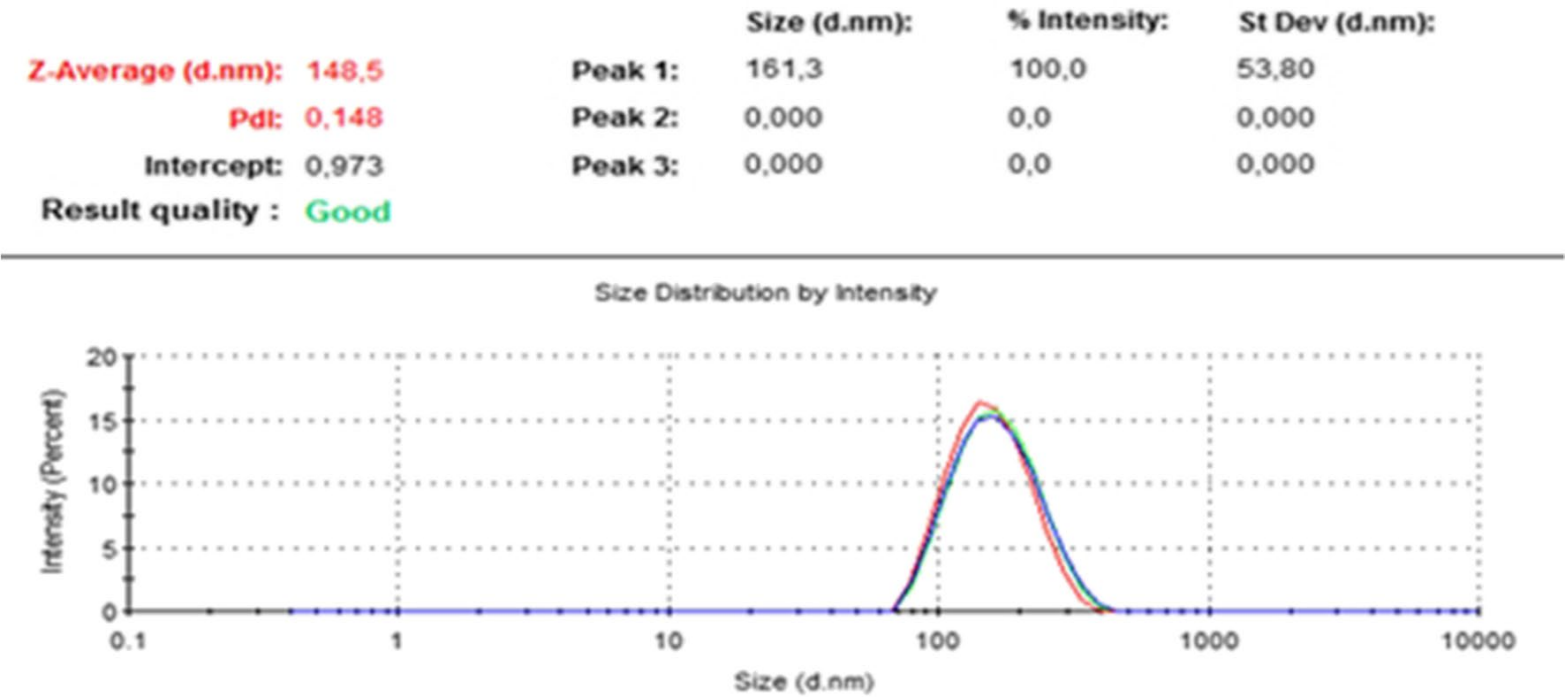
The particle size of MA-PLGA

**Fig. 7:**
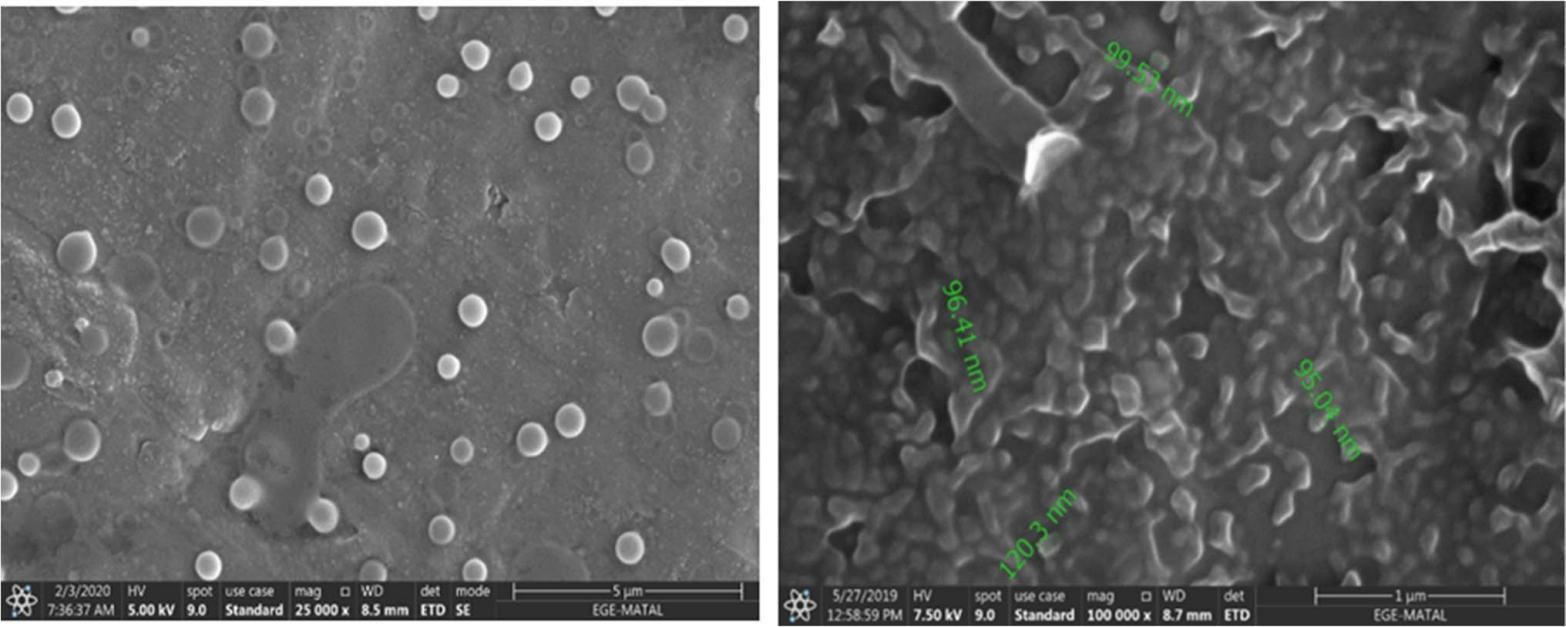
SEM images of MA-PLGA

**Fig. 8:**
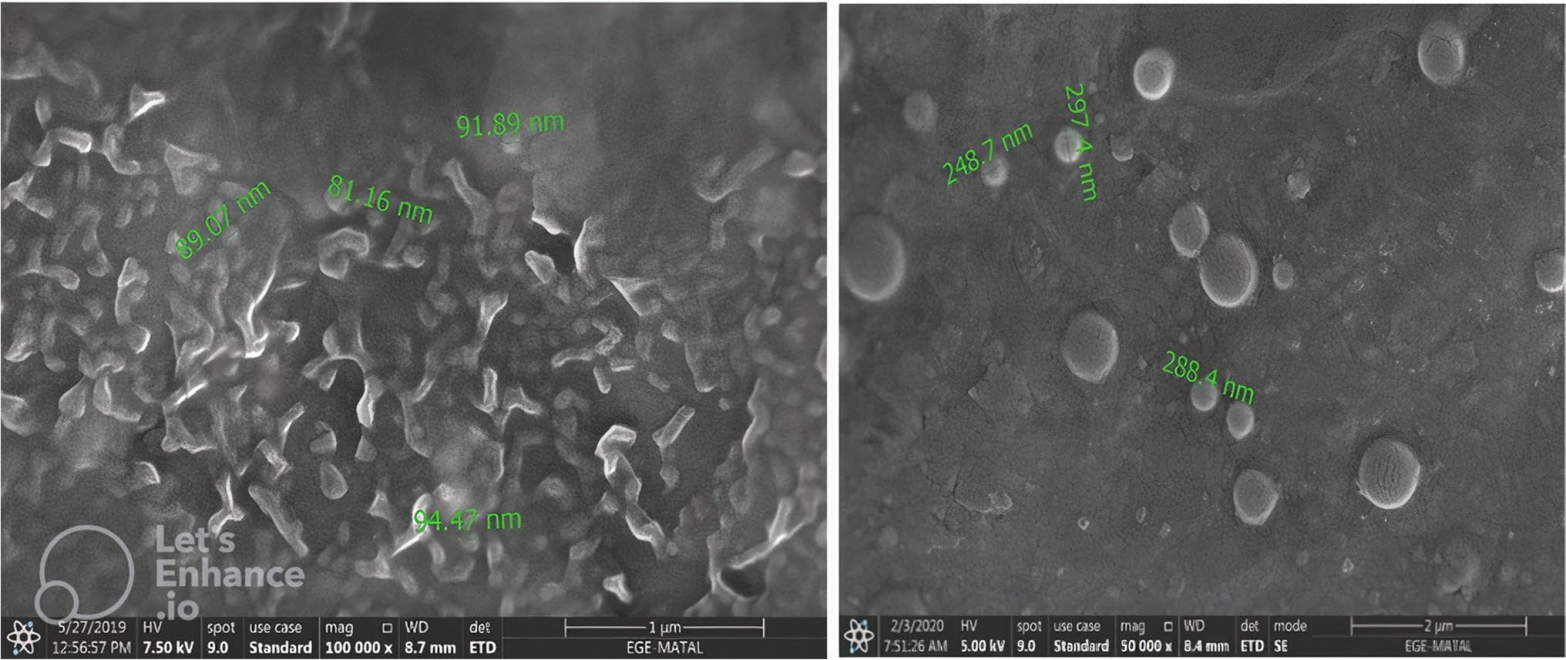
SEM images of MA-L-DOPA-PLGA

Encapsulation studies were evaluated by analyzing DLS analysis and SEM images together. The encapsulation process was optimized. When the studies were first started, the nanoparticle sizes obtained in the DLS results for the L-DOPA-PLGA compound were on average 3915 nm, while these dimensions were reduced to 123 nm **(**Fig. 5**)** with the method obtained by optimizing the conditions through various variations. When the particle sizes obtained are evaluated accordingly, the optimization process has been successful, and the method has been finalized. The nanoparticle size of MA-PLGA (Figs. 6 and 7) was found as 148.5 nm in DLS analysis. In SEM images (Fig. 8) of MA-L-DOPA-PLGA, the nanoparticle size changes between 248 and 298 nm.

### Radiolabeling

The radiochemical yields of radiolabeled compounds obtained because of (TLRC) analysis are given in Table 2. Therefore, when considered as a whole, it is seen that the radiochemical yields obtained are above 95% except [^99m^Tc]Tc-L-DOPA-PLGA and have the potential to be used in nuclear medicine. Retention times of radiolabelled compounds in HPLRC are given retention times (R_t_) of [^99m^Tc]TcO_4_^−^, [^99m^Tc]Tc-MA, [^99m^Tc]Tc-L-DOPA, [^99m^Tc]Tc-MA-L-DOPA, were 2.96, 3.19, 3.10, and 3.21 min, respectively.

**Table 2:** Relative front (R_f_) values of radiolabeled compounds

Radiolabeled compounds	R_f_ values	Radiolabeling efficiency (%) (n = 10)
Na^[99m^Tc]TcO_4_	0.87	–––
Reduced [^99m^Tc]Tc	0.03	–––
[^99m^Tc]Tc -MA	0.91	99.67 ± 0.60
[^99m^Tc]Tc -L-DOPA	0.83	99.15 ± 0.37
[^99m^Tc]Tc -MA-L-DOPA	0.90	99.19 ± 0.93
[^99m^Tc]Tc -MA-PLGA	0.88	99.38 ± 0.84
[^99m^Tc]Tc -L-DOPA-PLGA	0.83	93.83 ± 0.61
[^99m^Tc]Tc -MA-L-DOPA-PLGA	0.87	98.93 ± 1.16

### Stability Studies

The radiochemical yields of the [^99m^Tc]Tc-MA, [^99m^Tc]Tc-MA-PLGA [^99m^Tc]Tc-MA-L-DOPA-PLGA were over 95% until 4 h except [^99m^Tc]Tc-L-DOPA and [^99m^Tc]Tc-MA-L-DOPA.

### Lipophilicity (logP)

Experimental and theoretical lipophilicity (logP) values of radiolabeled compounds are demonstrated in Table 3. According to the Table 3, it was seen that [^99m^Tc]Tc-MA, [^99m^Tc]Tc-L-DOPA, [^99m^Tc]Tc-MA-PLGA, and [^99m^Tc]Tc-MA-L-DOPA-PLGA had a hydrophilic structure. [^99m^Tc]Tc-MA, [^99m^Tc]Tc-MA-PLGA, and [^99m^Tc]Tc-MA-L-DOPA-PLGA compounds range between -1 and + 1.5. Especially when the lipophilicity values of PLGA and encapsulated compounds are compared, it is thought that the PLGA structure has a positive effect on the increase of the lipophilicity of the compounds.

**Table 3:** Experimental lipophilicity values of radiolabeled compounds

Radiolabeled compounds	Theoretical log P	Experimental log P
[^99m^Tc]Tc-MA	1.88 ± 0.95	-0.60 ± 0.01
[^99m^Tc]Tc -L-DOPA	-0.22 ± 0.32	-1.59 ± 0.01
[^99m^Tc]Tc -MA-L-DOPA	–––––––	1.52 ± 0.01
[^99m^Tc]Tc -MA-PLGA	–––––––	-0.69 ± 0.28
[^99m^Tc]Tc -MA-L-DOPA-PLGA	–––––––	-0.25 ± 0.09

### Cytotoxicity Study

**Fig. 9:**
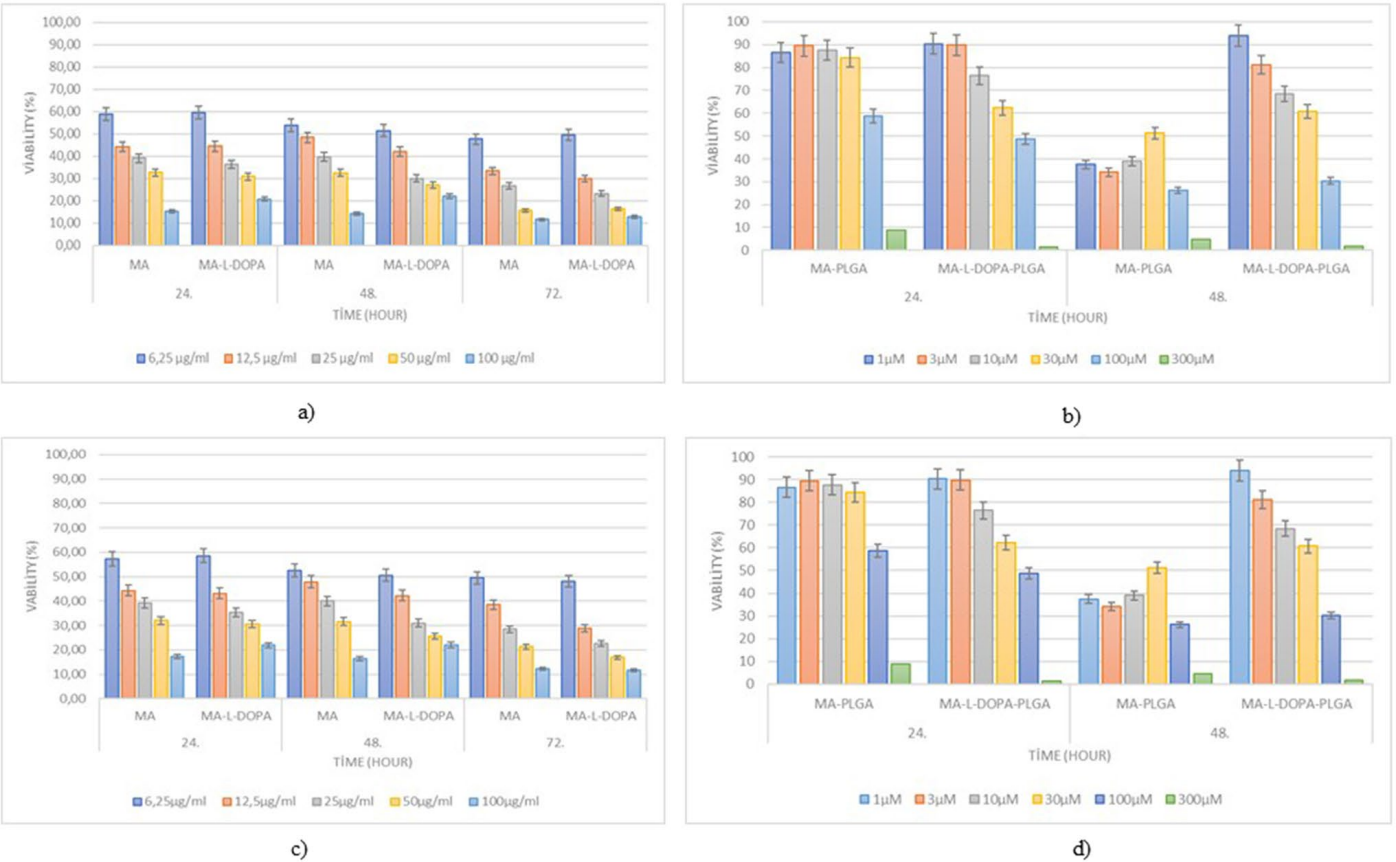
Percent viability values on compounds

The cell viability of MA and MA-L-DOPA compounds and encapsulated compounds (MA-PLGA and MA-L-DOPA-PLGA) on the SH-SY5Y and PC-12 cell lines are given in Fig. 9. It is seen that the cytotoxic effects of all substances on SH-SY5Y cells increase with increasing concentration values. According to Fig. 9 and Table 4, similar results emerge for MA-L-DOPA and MA-L-DOPA-PLGA compounds containing L-DOPA. The cytotoxic effect of the MA compound on the SH-SY5Y cell line is less than that of the MA-L-DOPA compound in Fig. 9. Comparing the % viability values of the MA compound and the MA-L-DOPA compound at the same concentration from the graphs given in Fig. 9, similar results were obtained, which can be explained by the fact that the MA molecule reduces the cytotoxic effect of the L-DOPA molecule. It is seen that the compounds encapsulated with PLGA show less cytotoxic effect at the same concentrations when MA and MA-L-DOPA molecules are compared to MA-PLGA and MA-L-DOPA-PLGA. It was observed that cytotoxicity increased on the PC-12 cell line with increasing concentrations. It is seen that the MA compound and MA-L-DOPA compounds have similar effects on the PC-12 cell line at the viability level. This is interpreted as the MA compound reducing the toxic effect of the L-DOPA compound. It is also considered a remarkable finding that the toxic effects of MA-PLGA and MA-L-DOPA-PLGA compounds are less than those of MA and MA-L-DOPA compounds.

**Table 4:** IC50 (µM) values of MA, MA-L-DOPA, MA-PLGA, and MA-L-DOPA-PLGA compounds on SH-SY5Y and PC-12 cell lines

IC_50_ (µM) value						
**Time (hour)**						
**Cell line**	**SH-SY5Y**			**PC-12**		
**Compound**	**24**	**48**	**72**	**24**	**48**	**72**
MA	12.94 ± 2.14	12.80 ± 2.24	7.45 ± 1.14	12.82 ± 2.21	12.63 ± 2.41	8.54 ± 1.37
MA-L-DOPA	12.89 ± 2.09	10.13 ± 2.08	7.16 ± 1.11	12.49 ± 2.21	10.03 ± 2.06	6.93 ± 1.09
MA-PLGA	55.51 ± 5.63	73.11 ± 2.34		105.3 ± 3.23	–––––-	
MA-L-DOPA-PLGA	86.79 ± 7.33	–––––		50.38 ± 14.96	31.25 ± 10.40	

### Incorporation Study

**Fig. 10:**
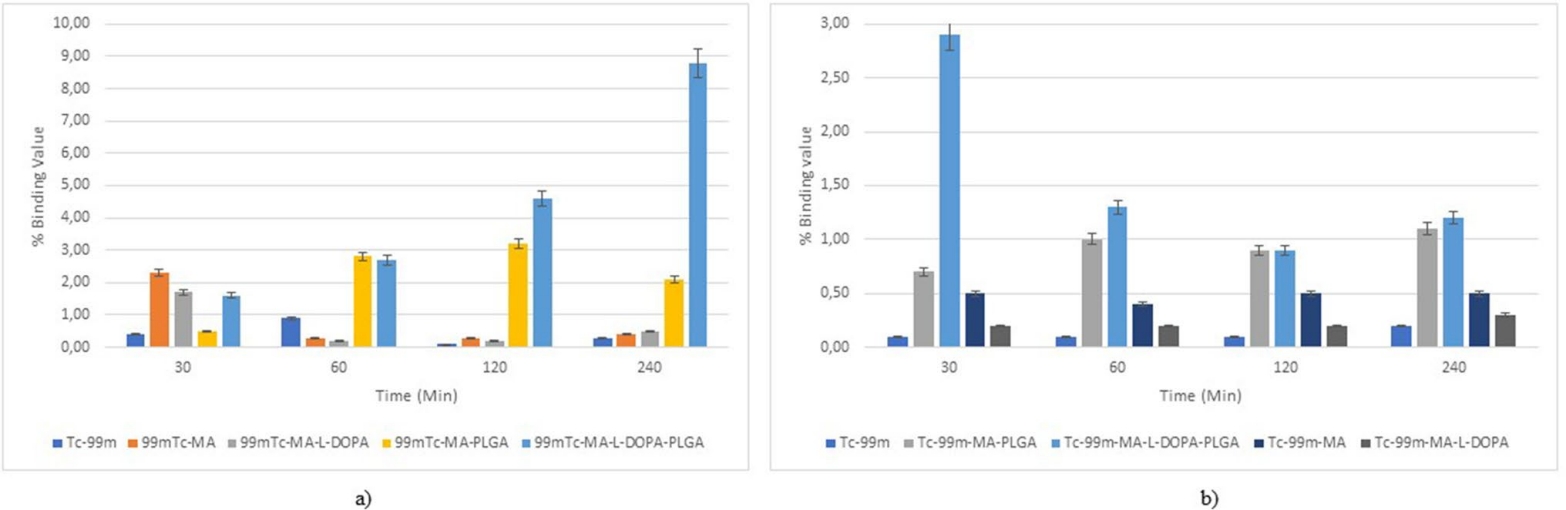
Changes in the % binding values of radiolabelled compounds

The binding values of MA and MA-L-DOPA compounds and encapsulated compounds (MA-PLGA and MA-L-DOPA-PLGA) on the SH-SY5Y and PC-12 cell lines are given in Fig. 10. On the SH-SY5Y cell line, it is observed that the % binding values of [^99m^Tc]Tc-MA and [^99m^Tc]Tc-MA-L-DOPA compounds decreased from 2.3 ± 0.8% to 0.5 ± 0.1% after 30 min. However, the % binding value of the [^99m^Tc]Tc-MA-PLGA compound encapsulated with PLGA increased from 0.5 ± 0.1% to 3.2 ± 0.8% after 30 min. The % binding values of the [^99m^Tc]Tc-MA-L-DOPA-PLGA compound increased from 1.6 ± 0.3% to 8.8 ± 0.8% after 30 min. When these two situations are compared, it can be thought that encapsulation with PLGA increases the uptake in the cell. When [^99m^Tc]Tc and radiolabelled compounds were compared, it was observed that [^99m^Tc]Tc-MA, [^99m^Tc]Tc-MA-L-DOPA, and [^99m^Tc]Tc-MA-L-DOPA-PLGA showed significantly (*p* < 0.05) higher uptake at 30 min. After the 30th minute, a significant difference is observed in the encapsulated [^99m^Tc]Tc-MA-PLGA and [^99m^Tc]Tc-MA-L-DOPA-PLGA compounds. On the PC-12 cell line, it is seen that the highest % binding value is in the [^99m^Tc]Tc-MA-L-DOPA-PLGA compound (2.90 ± 0.20%) at the 30th minute. In this compound, it is observed that the % binding values decrease up to 0.90 ± 0.10% after 30 min. When [^99m^Tc]Tc and radiolabelled compounds were compared, it was observed that [^99m^Tc]Tc-MA, [^99m^Tc]Tc-MA-L-DOPA, and [^99m^Tc]-MA-PLGA radiolabelled compounds did not show much uptake at 30 min (*p* > 0.05). The higher uptake in the [^99m^Tc]Tc-MA-L-DOPA-PLGA compound shows that encapsulation with PLGA increases uptake in the cell. When both cell lines are evaluated together, it can be said that the uptake values of PLGA-encapsulated compounds are statistically significant in SH-SY5Y and PC-12 cells.

### Biodistribution

**Fig. 11:**
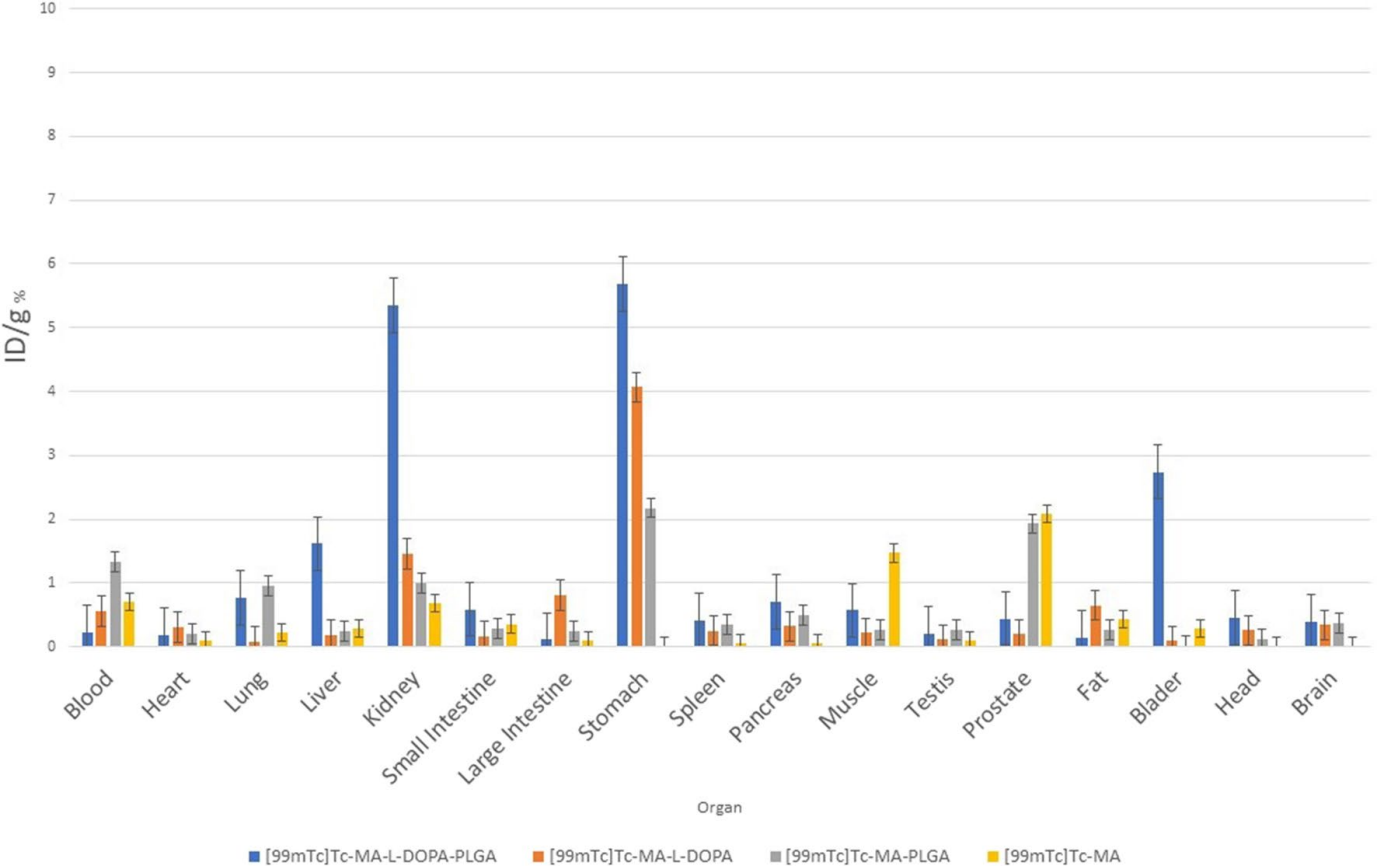
Time-dependent biodistribution of radiolabeled compounds in some organs in control group rats

**Fig. 12:**
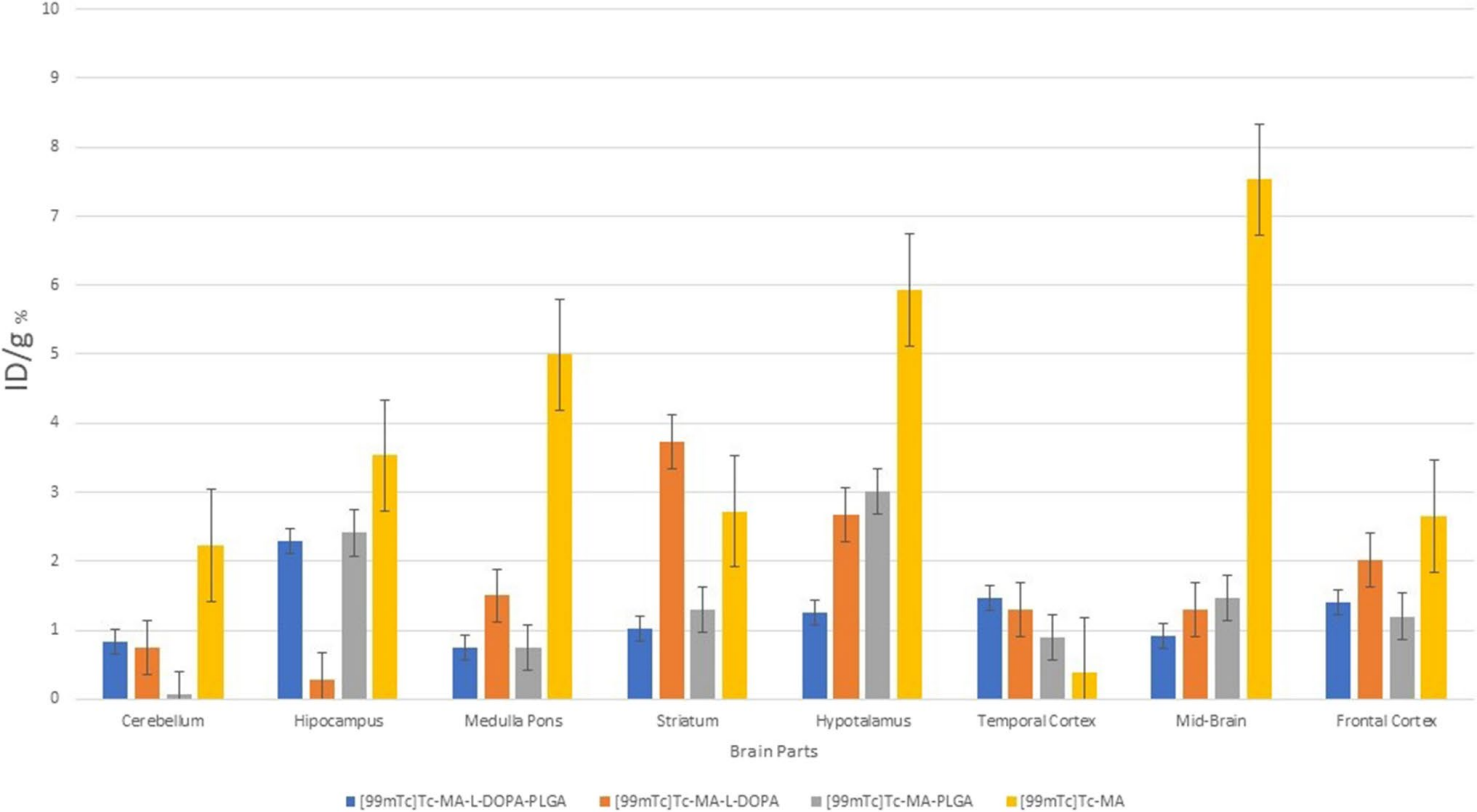
Time-dependent biodistribution of radiolabelled compounds in the brain of control group rats

**Fig. 13:**
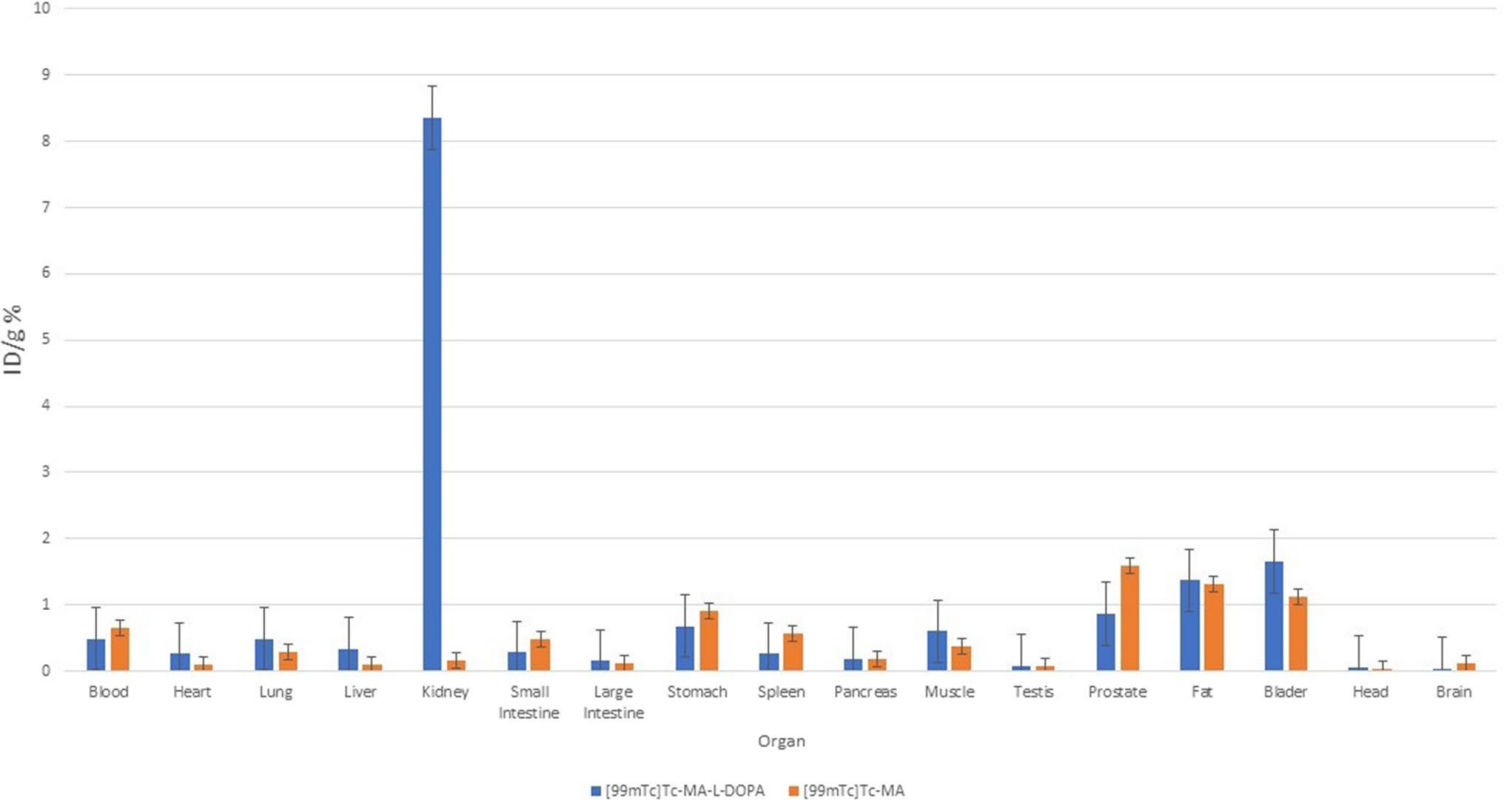
Time-dependent biodistribution of radiolabelled compounds in some organs in Parkinson’s model rats

It is seen that [^99m^Tc]Tc-MA is mostly retained in the prostate and muscle tissue among organs (Fig. 11). It is also retained in the kidneys, blood, fat, small intestine, and large intestine. The results of [^99m^Tc]Tc-L-DOPA and [^99m^Tc]Tc-MA-L-DOPA were compared, it was observed that the uptake was higher in the stomach (4.07 ± 0.19%) and kidney (1.46 ± 0.67%). When the encapsulated structures were analyzed, it was observed that [^99m^Tc]Tc-MA-L-DOPA-PLGA compound showed the highest uptake in stomach (5.35 ± 0.63%), kidney (5.68 ± 0.72%) and bladder (2.78 ± 0.65%). Especially when compared with [^99m^Tc]Tc-MA-L-DOPA, the uptake in these organs was significantly (p < 0.05) higher. When [^99m^Tc]Tc-MA and [^99m^Tc]Tc-MA-PLGA were compared, it was observed that [^99m^Tc]-MA-PLGA compound had higher uptake value in blood (1.34 ± 0.15%) and stomach (2.18 ± 0.22%) tissues, while there was no significant difference in other tissues. In general, PLGA-encapsulated compounds showed higher uptake in kidneys (5.35 ± 0.63%) and stomach (5.68 ± 0.72%) compared to nonencapsulated compounds. [^99m^Tc]Tc-MA was most highly retained in the midbrain, hypothalamus, medulla pons, hippocampus, striatum, frontal cortex, and cerebellum (Fig. 12). [^99m^Tc]Tc-MA is retained in the mid-brain (mid-brain) (7.53 ± 0.53%). On the other hand, its retention in the hippocampus, striatum, and frontal cortex, which are involved in dopamine pathways, is also significant. However, [^99m^Tc]Tc-MA-L-DOPA shows higher uptake (3.73 ± 0.49%) in the striatum. However, it is noteworthy that [^99m^Tc]Tc-MA showed a wider distribution than the dopaminergic system in the mechanism of uptake in the hypothalamus, medulla pons, and cerebellum. In order to understand the underlying mechanism of this wider distribution, we performed another biodistribution study on rats that had degeneration in the SNc with rotenone injection and named as Parkinson’s animal model in our study. Encapsulated compounds were not included in the second biodistribution study compared to other labelled compounds. According to Fig. 13, it is seen that [^99m^Tc]Tc-MA is mostly retained in the prostate (1.60 ± 0.67%), bladder (1.12 ± 0.56%), and fat (1.32 ± 0.35%) tissues. The stomach, spleen, blood, muscle, small intestine, and lung were also involved. When compared with control group data (Fig. 11), no significant difference (*p* > 0.05) was observed.

**Fig. 14:**
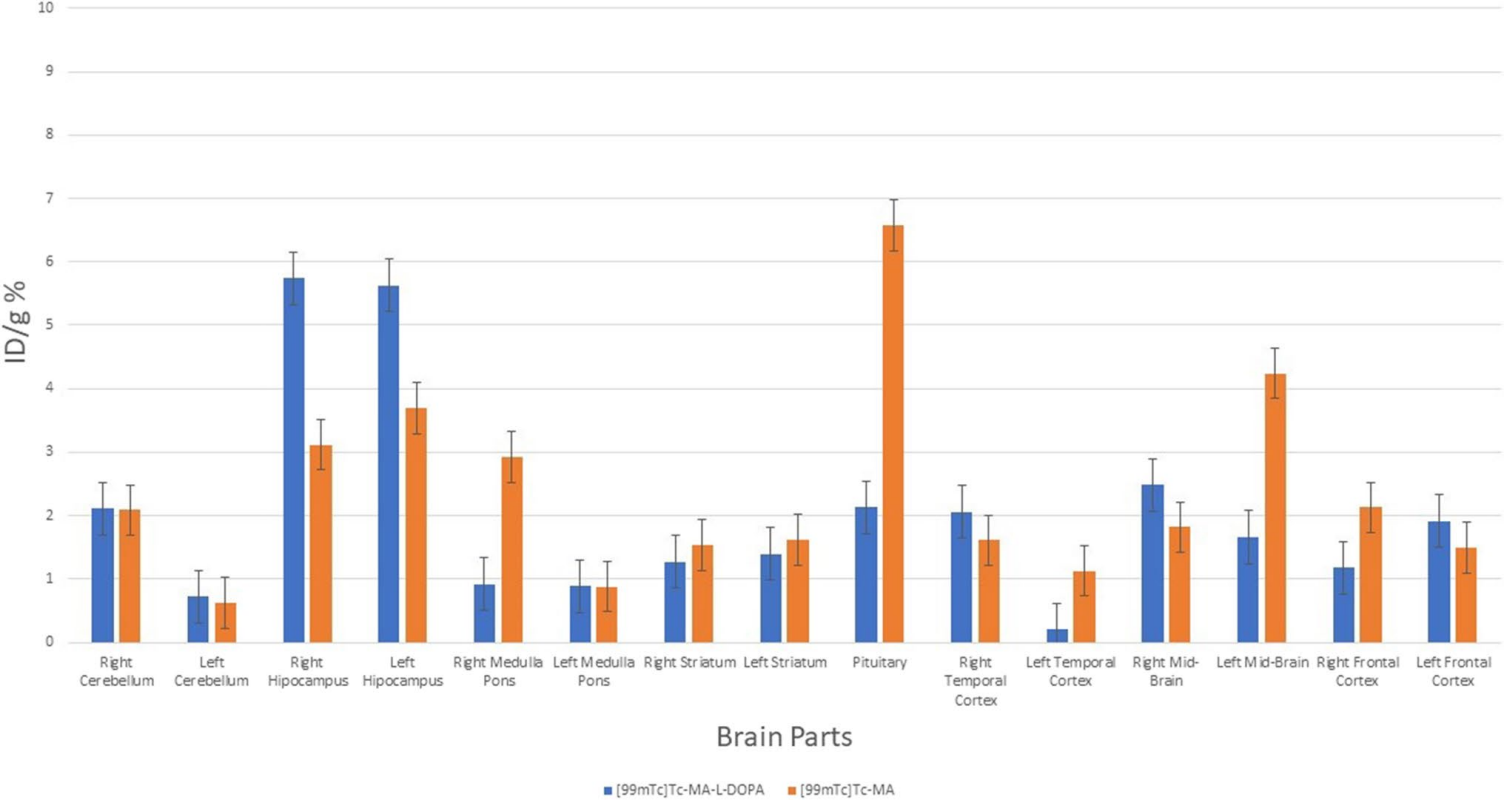
Time-dependent biodistribution of radiolabelled compounds in the brain of Parkinson’s model rats

When [^99m^Tc]Tc-MA-L-DOPA was analyzed, the highest uptake value was observed in the kidney (8.36 ± 0.85%) with a significant difference. On the other hand, significant uptake was also observed in bladder (1.66 ± 0.28%), fat (1.37 ± 0.17%), and prostate (0.87 ± 0.12%). When the results are compared with the control group data (Fig. 11), they are very similar. While analyzing the involvement in the brain parts, since the SNc part of the brain (AP: 5.2 mm, L: 2.4 mm, V: 8 mm) where stereostatic intervention was performed was in the right part of the brain and degeneration was performed there with rotenone injection, brain tissues were analyzed in two separate parts as right and left. Only the pituitary gland was analyzed instead of the hypothalamus and this tissue could not be divided into two parts; therefore, it was analyzed as a whole. From the control group data given in Fig. 12, it is seen that [^99m^Tc]Tc-MA is retained in the midbrain (7.537.53 ± 0.52%), pituitary, medulla pons, hippocampus, striatum, frontal cortex, and cerebellum. In Fig. 14, these parts are analyzed as left and right. When the midbrain was analyzed, we observed that the involvement in the left midbrain, in terms of [^99m^Tc]Tc-MA retainment (4.03 ± 0.32%) was higher. The striatum and hippocampus regions were more involved on the left side, but no significant difference was observed compared to the right side. Frontal and temporal cortexes were also more involved in the right hemisphere, but there was no significant difference between them and the left hemisphere. However, significantly more involvement was observed in the medulla pons and cerebellum than on the right side. When compared in terms of the cerebellum, the left cerebellum (1.02 ± 0.32%) and the cerebellum parts (0.97 ± 0.21%) show similar values (Fig. 12 and Fig. 14). However, there is a significant difference (*p* < 0.05) in favor of the increase in the right part (2.12 ± 0.25%).

## Discussion

As a result, the [^99m^Tc]-MA and encapsulated radiolabeled compounds are considered to be stable since they have a radiochemical yields above % 95 [43] as stated in the literature over time. Neurodegeneration is associated with dysfunction of the synapse, loss of neurons, neuron structure, neuronal functions, and the deposition of physiochemically altered variants of proteins in the brain. Neurodegeneration alters the proper functioning of the human brain. Neurodegeneration is a major health concern. Parkinson's disease and Alzheimer’s disease are the most common neurodegenerative diseases. xherapeutic approaches are symptomatic and do not reverse the disease process. New studies focus on therapeutics that target the pathogenesis of neurodegenerative diseases. Nanoparticles are used for targeted delivery into many organs, including the brain. Nanotherapeutics have a potential to cross the blood brain barrier and reverse neurodegeneration [44]. Liposomal and polymeric nanoparticles have been studied for targeted brain delivery due to the ease of surface modification with ligands and cell-penetrating peptides (CPPs) [45].

Parkinson’s disease, the second most common neurodegenerative disorder, lacks effective therapy targeting the pathogenesis of the disease. Medicine helps relieving motor and non-motor symptoms of the disease. As PD is caused by the loss of dopaminergic neurons in substantia nigra region of the brain, the principal goal of treatment is to replenish dopamine levels. There are several types of medications available to treat symptoms of PD: levodopa, dopamine agonists (apomorphine hydrochloride, pergolide, pramipexole dihydrochloride, ropinirole hydrochloride, and rotigotine), inhibitors of enzymes that inactivate dopamine (monoamine oxidase type B [MAO-B] inhibitors and catechol-O-methyl transferase [COMT] inhibitors), anticholinergic drugs, and amantadine. Levodopa is a precursor of dopamine that helps restore motor functions resulting from the loss of dopamine. Carbidopa is combined with levodopa to inhibit the peripheral breakdown of levodopa before it reaches the brain. Additionally, entacapone and tolcapone are also used to prevent methylation of levodopa through catechol-O-methyl transferase (COMT), thereby preventing levodopa loss through methylation.

Current therapy in neurodegenerative disorders control the symptoms and clinical progression rather than eliminating the cause of the disease. To date, disruption of the blood–brain barrier in in patients and animal models has been minimally evaluated. The blood brain barrier not only plays a role in the pathogenesis of PD, but also interferes with the efficacy of PD medications. Present medications have limited benefit due to the inability to transport sufficient doses to the brain through the blood–brain barrier. Most drugs have poort permeability though the barrier [46]. These factors highlight the importance of early diagnosis of PD, preferably in the prodromal or pre-motor phase. Early diagnosis may be considered in patients with early features such as neck pain, shoulder pain, neuropsychiatric features such as depression anxiety, hypotension, dizziness, sexual dysfunction, neurogenic bladder, sleep disturbances (insomnia, REM sleep behavior disorder, parasomnia), restless leg syndrome, hyposmia, and unspecified pain [47]. Experimental and theoretical lipophilicity (logP) values of radiolabeled compounds are demonstrated in Table 3. In neurological research, the capacity of a compound to permeate the BBB (Blood–Brain Barrier) is contingent upon various factors. The lipid solubility of the compound, its protein-binding affinity, molecular size, and charge can significantly influence this permeability. If the logP values obtained in the research are between -1 and + 1, it has been reported that the studied compound has a BBB permeability that increases linearly in this range and varies from 70 to 100% [48–50]. According to Table 3, it was seen that [^99m^Tc]Tc-MA, [^99m^Tc]Tc-L-DOPA, [^99m^Tc]Tc-MA-PLGA, and [^99m^Tc]Tc-MA-L-DOPA-PLGA had a hydrophilic structure. When the values are compared with the ranges in the literature [48–50], it is seen that [^99m^Tc]Tc-MA, [^99m^Tc]Tc-MA-PLGA, and [^99m^Tc]Tc-MA-L-DOPA-PLGA compounds range between -1 and + 1.5. Especially when the lipophilicity values of PLGA and encapsulated compounds are compared, it is thought that the PLGA structure has a positive effect on the increase of the lipophilicity of the compounds. It is also known from the literature that PLGA increases lipophilicity [51].

The cytotoxic effects of all MA-PLGA and MA-L-DOPA-PLGA on SH-SY5Y cells increase with increasing concentration values. There are studies showing that L-DOPA and its derivatives have cytotoxic effects on the SH-SY5Y cell line [52–54]. In these studies, it is observed that the cytotoxic effects of L-DOPA and its derivatives on the SH-SY5Y cell line increase with increasing concentrations. In addition, IC-50 values of compounds were calculated on the SH-SY5Y and PC-12 cell lines and given in Table 4. According to Fig. 9 and Table 4, similar results emerge for MA-L-DOPA and MA-L-DOPA-PLGA compounds containing L-DOPA. In the studies conducted on SH-SY5Y cell lines, there is some research showing that the MA compound reduces the cytotoxic effect caused by free oxygen radicals on the cell line [19, 25, 55, 56]. Figure 9 is examined from this perspective, it is seen that the cytotoxic effect of the MA compound on the SH-SY5Y cell line is less than that of the MA-L-DOPA compound. This situation shows that the L-DOPA molecule has a cytotoxic effect with an increasing concentration on the SH-SY5Y cell line [52–54, 56], but the cytotoxic effect of the MA-L-DOPA molecule is caused by free oxygen radicals. Comparing the % viability values of the MA compound and the MA-L-DOPA compound at the same concentration from the graphs given in Fig. 9, similar results were obtained, which can be explained by the fact that the MA molecule reduces the cytotoxic effect of the L-DOPA molecule. It is seen that the compounds encapsulated with PLGA show less cytotoxic effect at the same concentrations when MA and MA-L-DOPA molecules are compared to MA-PLGA and MA-L-DOPA-PLGA. This can be explained by the fact that the PLGA structure is biodegradable [29, 32, 34]. It was observed that cytotoxicity increased on the PC-12 cell line with increasing concentrations. There are studies in the literature showing that the L-DOPA molecule has a cytotoxic effect at high concentrations in the PC-12 cell line [57–59]. It is seen that the MA compound and MA-L-DOPA compounds have similar effects on the PC-12 cell line at the viability level. This is interpreted as the MA compound reducing the toxic effect of the L-DOPA compound. It is also considered a remarkable finding that the toxic effects of MA-PLGA and MA-L-DOPA-PLGA compounds are less than those of MA and MA-L-DOPA compounds. This situation can be explained by the fact that the PLGA structure is biodegradable [29, 32].

On the SH-SY5Y cell line, it is observed that the % binding values of [^99m^Tc]Tc-MA and [^99m^Tc]Tc-MA-L-DOPA compounds decreased from 2.3 ± 0.8% to 0.5 ± 0.1% after 30 min. However, the % binding value of the [^99m^Tc]Tc-MA-PLGA compound encapsulated with PLGA increased from 0.5 ± 0.1% to 3.2 ± 0.8% after 30 min. The % binding values of the [^99m^Tc]Tc-MA-L-DOPA-PLGA compound increased from 1.6 ± 0.3% to 8.8 ± 0.8% after 30 min. When these two situations are compared, it can be thought that encapsulation with PLGA increases the uptake in the cell. It is thought that the increased uptake of PLGA-encapsulated compounds is due to the biocompatibility of PLGA [32]. When [^99m^Tc]Tc and radiolabelled compounds were compared, it was observed that [^99m^Tc]Tc-MA, [^99m^Tc]Tc-MA-L-DOPA, and [^99m^Tc]Tc-MA-L-DOPA-PLGA showed significantly (*p* < 0.05) higher uptake at 30 min. After the 30th minute, a significant difference is observed in the encapsulated [^99m^Tc]Tc-MA-PLGA and [^99m^Tc]Tc-MA-L-DOPA-PLGA compounds. On the PC-12 cell line, it is seen that the highest % binding value is in the [^99m^Tc]Tc-MA-L-DOPA-PLGA compound (2.90 ± 0.20%) at the 30th minute. In this compound, it is observed that the % binding values decrease up to 0.90 ± 0.10% after 30 min. When [^99m^Tc]Tc and radiolabelled compounds were compared, it was observed that [^99m^Tc]Tc-MA, [^99m^Tc]Tc-MA-L-DOPA, and [^99m^Tc]-MA-PLGA radiolabelled compounds did not show much uptake at 30 min (*p* > 0.05). The higher uptake in the [^99m^Tc]Tc-MA-L-DOPA-PLGA compound shows that encapsulation with PLGA increases uptake in the cell. When both cell lines are evaluated together, it can be said that the uptake values of PLGA-encapsulated compounds are statistically significant in SH-SY5Y and PC-12 cells. Similar results can be seen in the study [60] examined the uptake of [^99m^Tc]Tc, [^99m^Tc]Tc-H, [^99m^Tc]Tc-BH and [^99m^Tc]Tc-BH-PLGA compounds on DAOY, U87-MG, and A-549 cells and found that the highest uptake was seen in [^99m^Tc]Tc-BH-PLGA compound in all cell lines. It was thought that this situation was caused by PLGA used as the encapsulation material. Based on this, it is thought that PLGA-encapsulated compounds show more uptake on the SH-SY5Y and PC-12 lines, and this is due to the biocompatibility of PLGA stated in the literature [32].

[^99m^Tc]Tc-MA is mostly retained in the prostate and muscle tissue among organs (Fig. 11). It is also retained in the kidneys, blood, fat, small intestine, and large intestine. In the literature, there are studies on the biodistribution of MA in rats [61, 62]. In their study on Sprague–Dawley rats, Leng et al. [61] reported that the MA compound was widely distributed in liver, kidney, heart, spleen and lung, and the uptake in liver and kidney was higher. As a reason for this, they stated that MA is distributed with blood, and especially blood flow and perfusion rates are the key factors affecting biodistribution. Another study was conducted by [62]. The distribution of Na[^99m^Tc]TcO4 (Sodium Pertechnetate) was investigated in Albion-Wistar rats 1 h after administration of Centella Asiatica (CA) plant extract in which MA is the main component. They showed that acute treatment with CA extract significantly (*p* < 0.05) changed the biodistribution of Na[^99m^Tc]TcO4 (Sodium Pertechnetate) in various organs and tissues (spleen, heart, duodenum, stomach, liver, muscle, kidney, testis and blood) except the brain. When these studies [61, 62] and Fig. 11 are evaluated together, it is seen that the results are similar. The results of [^99m^Tc]Tc-L-DOPA and [^99m^Tc]Tc-MA-L-DOPA were compared, it was observed that the uptake was higher in the stomach (4.07 ± 0.19%) and kidney (1.46 ± 0.67%). Studies show that L-DOPA is metabolized in the gastrointestinal tract, kidney, and liver [63]. This is considered to be the reason for the higher uptake of [^99m^Tc]Tc-MA-L-DOPA conjugated with L-DOPA in stomach and kidney. When the encapsulated structures were analyzed, it was observed that [^99m^Tc]Tc-MA-L-DOPA-PLGA compound showed the highest uptake in stomach (5.35 ± 0.63%), kidney (5.68 ± 0.72%) and bladder (2.78 ± 0.65%). Especially when compared with [^99m^Tc]Tc-MA-L-DOPA, the uptake in these organs was significantly (*p* < 0.05) higher. When [^99m^Tc]Tc-MA and [^99m^Tc]Tc-MA-PLGA were compared, it was observed that [^99m^Tc]-MA-PLGA compound had higher uptake value in blood (1.34 ± 0.15%) and stomach (2.18 ± 0.22%) tissues, while there was no significant difference in other tissues. In general, PLGA-encapsulated compounds showed higher uptake in kidneys (5.35 ± 0.63%) and stomach (5.68 ± 0.72%) compared to nonencapsulated compounds. This situation is related to biodegradable PLGA. The PLGA structure is intensively retained in the liver, kidney, and brain in drug delivery systems using PLGA [64]. In our study, [^99m^Tc]Tc-MA was most highly retained in the midbrain, hypothalamus, medulla pons, hippocampus, striatum, frontal cortex, and cerebellum (Fig. 12). [^99m^Tc]Tc-MA retention was notably increased in the midbrain. This may be attributed to the fact that dopaminergic neurons are densely located in the SNc section and ventral tegmental area in the midbrain [65]. The three main pathways in the brain in which dopamine is involved are nigrostriatal, mesolimbic, and mesocortical pathways. The nigrostriatal pathway provides dopaminergic input from the SNc to the dorsal striatum and is often associated with the formation of automatic stimulus–response connections such as motor control and habits. The mesolimbic pathway projectsfrom the ventral tegmental area (VTA) to the nucleus accumbens (NAcc) where it is transmitted to the limbic system in the ventral striatum, i.e., the amygdala, hippocampus, and prefrontal cortex (PFC). The mesocortical pathway provides direct dopaminergic input from the VTA to the frontal cortex [65, 66]. In the results Our results revealed that [^99m^Tc]Tc-MA is retained in the mid-brain (mid-brain) (7.53 ± 0.53%). On the other hand, its retention in the hippocampus, striatum, and frontal cortex, which are involved in dopamine pathways, is also significant. However, [^99m^Tc]Tc-MA-L-DOPA shows higher uptake (3.73 ± 0.49%) in the striatum. However, it is noteworthy that [^99m^Tc]Tc-MA showed a wider distribution than the dopaminergic system in the mechanism of uptake in the hypothalamus, medulla pons, and cerebellum. In order to understand the underlying mechanism of this wider distribution, we performed another biodistribution study on rats that had degeneration in the SNc with rotenone injection and named as Parkinson's animal model in our study. Encapsulated compounds have been reported to be more effective in drug transport studies in brain research [29, 32, 67–69]. The lower uptake of MA and MA-L-DOPA compounds, despite expectations of higher efficacy, suggests that these compounds might require additional surface modifications. A study by Li and Sabliov [69] indicated that compounds encapsulated with PLGA and PLA struggle to traverse the BBB without appropriate modifications. Moreover, in their research, PLGA and PLA-coated nanoparticles were administered to rats or mice either orally, via the carotid artery, or intravenously. The unmodified PLGA/PLA nanoparticles displayed a markedly low (< 1%) brain uptake. They also stated that PLGA nanoparticles with surface modifications such as surface coated and addition of targeting ligands improved the brain delivery of drugs compared to unmodified PLGA nanoparticles. It was stated that the ligands covalently bound to the surface of the particles were the effect that most improved the brain uptake of nanoparticles. From this point of view, it is thought that the encapsulated compounds used in the study ([^99m^Tc]Tc-MA-PLGA and [^99m^Tc]Tc-MA-L-DOPA-PLGA) should be modified. It is predicted that these compounds will penetrate more into brain by optimizing these compounds in future studies. However, encapsulated compounds were not included in the second biodistribution study compared to other labelled compounds. According to Fig. 13, it is seen that [^99m^Tc]Tc-MA is mostly retained in the prostate (1.60 ± 0.67%), bladder (1.12 ± 0.56%), and fat (1.32 ± 0.35%) tissues. The stomach, spleen, blood, muscle, small intestine, and lung were also involved. When compared with control group data (Fig. 11), no significant difference (*p* > 0.05) was observed. The results are in parallel with studies in the literature [61, 62] involving the biodistribution of MA compound in rats. When [^99m^Tc]Tc-MA-L-DOPA was analyzed, the highest uptake value was observed in the kidney (8.36 ± 0.85%) with a significant difference. On the other hand, significant uptake was also observed in bladder (1.66 ± 0.28%), fat (1.37 ± 0.17%) and prostate (0.87 ± 0.12%). When the results are compared with the control group data (Fig. 11), they are very similar. The intense uptake in the kidney is attributable to the metabolism of L-DOPA in the kidney [63]. While analyzing the involvement in the brain parts, since the SNc part of the brain (AP: 5.2 mm, L: 2.4 mm, V: 8 mm) where stereostatic intervention was performed was in the right part of the brain and degeneration was performed there with rotenone injection, brain tissues were analyzed in two separate parts as right and left. Only the pituitary gland was analyzed instead of the hypothalamus and this tissue could not be divided into two parts; therefore, it was analyzed as a whole. From the control group data given in Fig. 12, it is seen that [^99m^Tc]Tc-MA is retained in the midbrain ( 7.537.53 ± 0.52%), pituitary, medulla pons, hippocampus, striatum, frontal cortex and cerebellum. In Fig. 14, these parts are analyzed as left and right. When the midbrain was analyzed, we observed that the involvement in the left midbrain, in terms of [^99m^Tc]Tc-MA retainment(4.03 ± 0.32%) was higher. This is thought to be due to the decrease in the density of dopaminergic neurons in the right midbrain with degeneration of the SNc. In terms of involvement, similar results were observed in both parts. The striatum and hippocampus regions were more involved on the left side, but no significant difference was observed compared to the right side. This is attributed to the lack of damage to dopaminergic neurons in these tissues during stereotaxic intervention. Frontal and temporal cortexes were also more involved in the right hemisphere, but there was no significant difference between them and the left hemisphere. However, significantly more involvement was observed in the medulla pons and cerebellum than on the right side. When compared in terms of the cerebellum, the left cerebellum (1.02 ± 0.32%) and the cerebellum parts (0.97 ± 0.21%) show similar values (Fig. 12 and Fig. 14). However, there is a significant difference (*p* < 0.05) in favor of the increase in the right part (2.12 ± 0.25%). Cerebellum plays a critical role in many motor and cognitive functions in the brain. Dopamine is thought to play a role in certain cerebellar functions [8–10]. However, it has been determined that tyrosine hydroxylase, the rate-limiting enzyme in catecholamine biosynthesis, is more prominent. There are studies suggesting that the dopamine produced is the precursor of adrenaline [8]. From this point of view, it leads to the conclusion that [^99m^Tc]Tc-MA should be examined in more detail in relation to dopamine and other catecholamines (adrenaline-epinephrine) in which dopamine is involved.

## Conclusion

Within the scope of the study, the MA-L-DOPA molecule was synthesized by conjugation of the MA molecule and L-DOPA molecule. Melting Point Determination, LC–MS, NMR, and FTIR analyses were performed to confirm the structure analysis of the synthesis. The quality control of the reaction was also supported by TLC and HPLC analyses. It was verified that the synthesis, in which all analyses were evaluated together, took place. Encapsulation of MA, L-DOPA, and MA-L-DOPA molecules with PLGA was performed. SEM images were taken after performing a DLS analysis of the structures obtained. As a result of the analysis, it was seen that encapsulation took place. The radiolabeling studies of MA, L-DOPA, MA-L-DOPA, MA-PLGA, L-DOPA-PLGA, and MA-L-DOPA-PLGA molecules with [^99m^Tc]Tc were performed. Quality controls for radiolabeling studies were carried out using TLRC and HPLRC methods. Radiolabeling efficiencies were found to be over 95%. Stability and lipophilicity determinations of radiolabeled compounds were made. It has been determined that radiolabeled compounds are stable and have the potential to cross the BBB. Within the scope of in vitro studies, cytotoxicity studies of inactive MA, MA-L-DOPA, MA-PLGA, and MA-L-DOPA-PLGA molecules were carried out. It was observed that the cytotoxicity of all compounds increased with concentration and the dose amount to be studied in living tissue was determined. Incorporation studies of radiolabeled [^99m^Tc]Tc-MA, [^99m^Tc]Tc-MA-L-DOPA, [^99m^Tc]Tc-MA-PLGA, and [^99m^Tc]Tc-MA-L-DOPA-PLGA on cell lines were also performed. It was determined that these compounds showed uptake in SH-SY5Y and PC-12 cell lines. In order to investigate the bioactivity of [^99m^Tc]Tc radiolabeled compounds, biodistribution studies were carried out on healthy male Sprague Dawley rats and Parkinson’s experimental model created by stereotaxic intervention. The data obtained revealed that [^99m^Tc]Tc-MA and [^99m^Tc]Tc-MA-L-DOPA have the potential to be used in the diagnosis of Parkinson's disease, but more detailed studies are recommended for in vitro and in vivo. To summarize, plant-based encapsulated nanoparticles are potential candidates for the early diagnosis and studies are required to distribute the effect of nanopharmaceuticals as a potential treatment in PD.

## Data Availability

The datasets in the current study are available from the corresponding author upon reasonable request.
